# Installation of metal clusters adjacent to dual-Fe sites for enhanced oxygen reduction

**DOI:** 10.1093/nsr/nwaf356

**Published:** 2025-08-28

**Authors:** Ming Liu, Xuemin Wang, Shoufu Cao, Xiaoqing Lu, Wei Li, Na Li, Xian-He Bu

**Affiliations:** School of Materials Science and Engineering, Tianjin Key Laboratory of Metal and Molecule-Based Material Chemistry, Nankai University, Tianjin 300350, China; State Key Laboratory of Elemento-Organic Chemistry, Frontiers Science Center for New Organic Matter, College of Chemistry, Nankai University, Tianjin 300071, China; School of Materials Science and Engineering, Tianjin Key Laboratory of Metal and Molecule-Based Material Chemistry, Nankai University, Tianjin 300350, China; School of Materials Science and Engineering, China University of Petroleum, Qingdao 266580, China; School of Materials Science and Engineering, China University of Petroleum, Qingdao 266580, China; School of Materials Science and Engineering, Tianjin Key Laboratory of Metal and Molecule-Based Material Chemistry, Nankai University, Tianjin 300350, China; School of Materials Science and Engineering, Tianjin Key Laboratory of Metal and Molecule-Based Material Chemistry, Nankai University, Tianjin 300350, China; State Key Laboratory of Elemento-Organic Chemistry, Frontiers Science Center for New Organic Matter, College of Chemistry, Nankai University, Tianjin 300071, China; School of Materials Science and Engineering, Tianjin Key Laboratory of Metal and Molecule-Based Material Chemistry, Nankai University, Tianjin 300350, China; State Key Laboratory of Elemento-Organic Chemistry, Frontiers Science Center for New Organic Matter, College of Chemistry, Nankai University, Tianjin 300071, China

**Keywords:** metal-organic frameworks, dual-Fe sites, atomic clusters, oxygen reduction reaction

## Abstract

Fe-based dual-metal center catalysts, featuring intermetallic *d-d* orbital coupling, have garnered significant interest for enhancing the oxygen reduction reaction (ORR). Modulation of the adjacent microenvironment to dual-metal sites is central to further enhancing the ORR performance, but remains an underexplored area. In this study, an atomic-scale electrocatalyst, consisting of dual-Fe sites and Fe-based atomic clusters (Fe_DS/MC_-NC), is synthesized. Driven by these Fe-based atomic clusters, Fe_DS/MC_-NC achieves a half-wave potential of up to 0.920 V vs reversible hydrogen electrode (RHE) during the ORR and exhibits a kinetic current density up to 7 times that of commercial Pt/C at 0.880 V vs RHE. Additionally, Fe_DS/MC_-NC achieves a maximum power density of 214.6 mW cm^−2^ and maintains a negligible expansion of the voltage window after >1000 cycles for Zn-air batteries. Theoretical calculations reveal that the Fe_3_O_4_ clusters contribute significantly to the dual-Fe sites in Fe_DS/MC_-NC. These Fe_3_O_4_ clusters, positioned adjacent to the dual-Fe sites, induce spin polarization of the active centers through a weak interaction. This results in a positive shift of the spin-down orbitals toward the vicinity of the Fermi energy level, enhancing the adsorption of the key reaction intermediate OOH* and ultimately accelerating the reaction kinetics. This work may open up an avenue to construct dual-metal center catalysts with tunable atomic structures.

## Teaser

In an iron-based atomic-scale electrocatalyst, the atomic clusters located near the double iron sites effectively regulate the reaction behavior of key reaction intermediates.

## INTRODUCTION

Portable clean energy systems are anticipated to meet society's increasing production demands, making the development of high-performance energy storage and conversion devices critical [1–5]. Metal-air batteries, known for their low cost, high safety, and impressive theoretical specific energy, have garnered significant attention [6,7]. A key factor in their operational efficiency is the selection of an appropriate oxygen reduction reaction (ORR) electrocatalyst. Commercial Pt/C catalysts, with their high position of *d*-band center near the Fermi energy level, exhibit strong adsorption of oxygen-containing intermediates [8–10]. This characteristic, combined with their prohibitive cost and low kinetic efficiency, makes them less desirable. Metal-nitrogen co-doped carbon (M-N-C), particularly Fe-N-C, is considered one of the most promising alternatives in high-efficiency ORR applications [11–16]. The active Fe-N*_x_* center in Fe-N-C features a distinctive *d*-band electronic structure, which is conducive to the regulation of adsorption and desorption behavior for reaction intermediates.

The ORR activity of Fe-N-C is usually improved by optimizing the adsorption energies of the ORR intermediates (OOH*, O*, and OH*) for the active center. For example, introducing nonmetallic heteroatoms such as S and P can redistribute the local charge, leveraging the electronegativity differences and orbital interaction between atoms [17–20]. This approach helps optimize the adsorption and desorption energy barriers of ORR intermediates at the active sites [21–23]. Alternatively, embedding metal sites adjacent to Fe sites to form dual-metal center catalysts (DMCs) can significantly modulate the electronic structure of the Fe center through *d-d* hybridization, helping to resolve the issue of excessive OH* adsorption [24–27]. Besides, compared with Fe-N-C, DMCs can also easily alter the adsorption configuration of O_2_* and OOH*, promote O−O bond cleavage, and thus accelerate the reaction kinetics [28–30]. Consequently, DMCs are emerging as a new frontier in ORR.

**Scheme 1.**
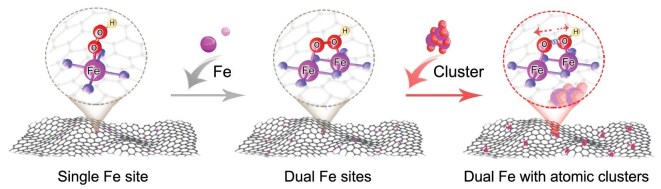
Schematic illustration of the interaction of Fe sites with OOH* under different coordination environments.

Considering the optimized adsorption of OH* and O_2_* by DMCs, fine-tuning OOH* adsorption through a more precise yet straightforward strategy could be a cipher to further enhancing ORR performance [21,28]. In previously reported single-atom systems, the construction of atomic-level clusters adjacent to the active sites is remarkably effective for the modulation of the electronic structure [31–38]. Similarly, implanting this concept into DMC systems is expected to further optimize the adsorption/desorption strength of reaction intermediates (in particular OOH*) at the active center through relatively weak interactions with the clusters, thereby maximizing the benefits of the bimetallic sites (Scheme 1). Unfortunately, the rational layout role of these clusters and their substantive impact on the electronic structure of active centers remain unclear in DMCs. The balance between the loss of active sites due to metal agglomeration during pyrolysis and the enhancement of catalytic performance by the metal clusters remains underexplored, with few reports addressing this issue. Therefore, on the basis of taking full advantage of DMCs, strategically positioning metal clusters adjacent to the bimetallic sites in DMCs is crucial yet challenging.

In light of these considerations, a dual-Fe site catalyst with Fe-based atomic clusters co-anchored in porous nitrogen-doped carbon (Fe_DS/MC_-NC) was developed via a one-step pyrolytic treatment. The Fe dimer was strategically employed as a precursor to ensure uniform configuration of dual-Fe sites in the target catalyst by confining its domain in a zeolitic imidazolate framework (ZIF-8). When Fe_DS/MC_-NC was used as an ORR catalyst, the half-wave potential increased by 17 mV, and the kinetic current doubled compared with a catalyst without atomic clusters. Furthermore, Fe_DS/MC_-NC demonstrated significantly enhanced energy density and cycling performance compared with commercial Pt/C when used as an air electrode catalyst in Zn-air batteries. Theoretical calculations indicate that the dual-Fe sites in Fe_DS/MC_-NC continue the dominant adsorption configuration for O_2_, and the Fe_3_O_4_ clusters induce pronounced spin polarization in the electronic structure at the Fe sites through a non-strongly correlated interaction. This shifts the spin-down orbitals near the Fermi energy level, promoting the adsorption of the key intermediate OOH* and thereby reducing the reaction energy barrier.

## RESULTS AND DISCUSSION

**Figure 1.**
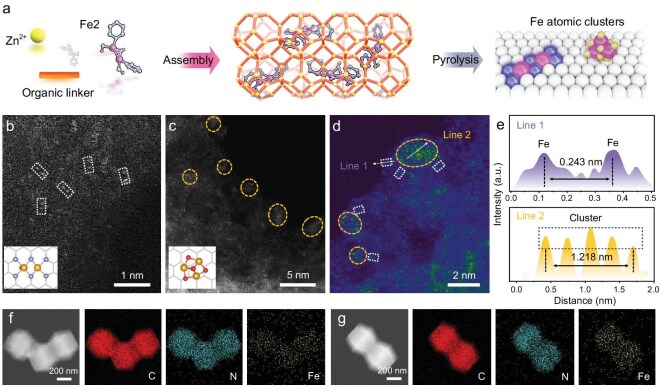
(a) Schematic illustration for synthesizing the Fe_DS/MC_-NC. AC-HAADF-STEM images of (b) Fe_DS_-NC and (c, d) Fe_DS/MC_-NC. (e) Intensity profiles obtained from the areas of Line 1 and Line 2 in (d). The TEM elemental mapping images of (f) Fe_DS_-NC and (g) Fe_DS/MC_-NC.

The preparation strategy for Fe_DS/MC_-NC involved a classical host-guest spatial restriction method, assisted by pyrolysis (Fig. 1a) [39,40]. In this approach, the ZIF-8, rich in nitrogen and easy to prepare, was used as the carbon substrate precursor, while cyclopentadienyliron dicarbonyl dimer (Fe2) served as the iron source to ensure pairwise dispersion of Fe sites within ZIF-8. Specifically, the effective dispersion of Fe2 in methanol was a decisive factor for its *in-situ* encapsulation within ZIF-8 cavities. By controlling the amount of Fe2 and assisted by simple room-temperature stirring, ZIF-8@Fe2-x (x = 5, 10, 15, 20, 40, and 80) was synthesized in a single step, in which x represents the mass (mg) of Fe2 added to the reaction. The powder X-ray diffraction (PXRD) pattern indicates that the initial crystal structure of ZIF-8 remains intact after the introduction of Fe2 (Fig. S1). Scanning electron microscopy (SEM) images also reveal negligible morphological changes in ZIF-8 following the *in-situ* encapsulation of Fe2 (Figs S2, S3). Additionally, the color of the sample shifted from pure white to a uniform light brown after the introduction of Fe2, confirming its even dispersion throughout the sample (Fig. S4). Fourier transform infrared spectra tests on the prepared samples revealed that the carbonyl signal originating from Fe2 still remained after *in-situ* coating of Fe2 into ZIF-8 (Fig. S5) [41]. The Fe-based carbon materials loaded on the nitrogen-doped carbon matrix substrate (Fe-NC-x) were then obtained through pyrolysis treatment. The PXRD analysis of Fe-NC-x reveals that, with a relatively small amount of Fe2 (x ≤15), only two diffraction peaks corresponding to graphitic carbon, ∼26° and 43°, are detected (Fig. S6). This suggests that the metals in the catalyst are likely in an atomically dispersed state. As the amount of Fe2 increases (x ≥20), peaks associated with Fe and Fe_3_O_4_ appear in the PXRD pattern, indicating the formation of metal clusters in the catalyst [42]. Meanwhile, transmission electron microscopy (TEM) further confirms that nanoparticles begin to attend unevenly in the target catalyst with the elevated Fe2 addition (Figs S7–S13). Obviously, a large number of nanoparticles could be observed for Fe-NC-80 (Fig. S13d).

**Figure 2.**
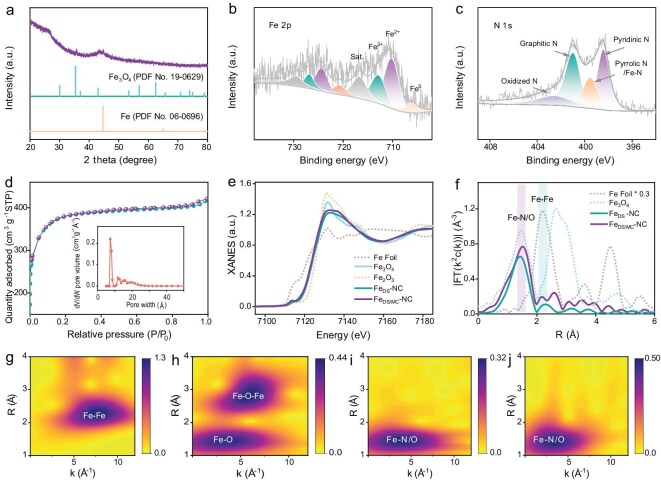
Structural characterization of Fe_DS/MC_-NC. (a) PXRD pattern, high resolution (b) Fe 2*p* and (c) N 1*s* XPS spectra, (d) nitrogen adsorption-desorption isotherms and pore size distribution of Fe_DS/MC_-NC. (e) Fe *K*-edge XANES spectra and (f) Fe *K*-edge Fourier transform (FT) spectra of samples. Wavelet transform (WT) of (g) Fe foil, (h) Fe_3_O_4_, (i) Fe_DS_-NC, and (j) Fe_DS/MC_-NC.

To facilitate differentiation, Fe-NC-15 (Fe_DS_-NC) and Fe-NC-40 (Fe_DS/MC_-NC) with and without clusters in the neighborhood of the dual-Fe sites were selected as representative samples to investigate in detail the different forms of metal dispersion. High-resolution TEM images show that most of the regions in Fe_DS/MC_-NC are similar to Fe_DS_-NC in that both lack visible nanoparticles, although a few nanoparticles are observed in Fe_DS/MC_-NC (Figs S10 and S12). Further analysis via aberration-corrected high-angle annular dark-field scanning transmission electron microscopy (AC-HAADF-STEM) reveals a significant difference between the two samples. As shown in Fig. 1b, Fe atoms are highly dispersed in pairs across the carbon matrix for Fe_DS_-NC, owing to the effective dispersion of Fe2 by the ZIF-8 structure in the precursor. In contrast, Fe_DS/MC_-NC contains abundant Fe-based atomic clusters surrounded by pairs of Fe atoms (Fig. 1c, d), mainly due to excess Fe promoting cluster formation through the Kirkendall effect during heat treatment [43]. Furthermore, the distance between the dual-Fe sites in Line 1 marked in Fig. 1d is ∼0.24 nm (Fig. 1e). This result is in agreement with our previous report. In addition, the metal spacing in Line 2 is ∼0.30 nm, which may be attributed to the (220) plane of Fe_3_O_4_ in combination with PXRD analysis of Fe_DS/MC_-NC (Fig. 2a). The above information confirms that the dispersion states of Fe in Fe_DS_-NC and Fe_DS/MC_-NC are fundamentally different, with dense atomic-level clusters added in the adjacent dual-Fe sites in Fe_DS/MC_-NC compared to the dual-Fe sites in Fe_DS_-NC.

The N_2_ adsorption and desorption isotherms indicate that NC, Fe_DS_-NC, and Fe_DS/MC_-NC exhibit typical Type I sorption isotherms and possess similar specific surface areas and pore size distributions (Figs 2d and S14). The presence of numerous microporous structures not only exposes effective catalytic sites but also accelerates mass transfer, thereby facilitating the catalytic process. Additionally, energy-dispersive X-ray spectroscopy (EDX) elemental mapping analyses show that Fe, N, and C are uniformly distributed in the samples, with Fe_DS/MC_-NC displaying a stronger cumulative signal for Fe compared with Fe_DS_-NC (Fig. 1f, g). Inductively coupled plasma mass spectrometry (ICP-MS) determined the Fe content in Fe_DS_-NC and Fe_DS/MC_-NC to be 1.1 wt% and 2.0 wt%, respectively (Table S1). The chemical composition of Fe_DS/MC_-NC was further examined using X-ray photoelectron spectroscopy (XPS). As shown in Fig. 2b, the Fe 2*p* spectrum of Fe_DS/MC_-NC reveals the presence of both Fe^2+^ (710.1 and 724.3 eV) and Fe^3+^ (712.8 and 726.9 eV) valence states, which arise from the dual-Fe sites and Fe_3_O_4_ within the carbon matrix. Additionally, weak signals at 706.1 and 721.2 eV are attributed to metallic Fe in Fe_DS/MC_-NC [42,44]. The weak Fe 2*p* signal due to the low Fe content in Fe_DS_-NC makes it difficult to analyze (Fig. S15). The N 1*s* spectra show that the nitrogen species in the samples mainly consist of pyridinic-N (398.4 eV), pyrrolic-N/Fe-N (399.8 eV), graphitic-N (401.0 eV), and oxidized-N (402.6 eV) (Figs 2c and S16) [11]. Furthermore, the high-resolution C 1*s* spectra were deconvoluted into three components: C-C (284.8 eV), C-N (285.7 eV), and C=O (288.9 eV) (Fig. S17) [3]. Raman spectroscopy was used to analyze the crystallinity of the carbon matrix substrate. The D-band, located ∼1350 cm^−1^, corresponds to defective carbon, while the G-band, ∼1580 cm^−1^, corresponds to graphitic carbon (Fig. S18) [45]. Compared with NC, the introduction of Fe2 increases the intensity ratios of the D and G bands (I_D_/I_G_) to 1.03 for Fe_DS_-NC and 1.11 for Fe_DS/MC_-NC, respectively. The higher I_D_/I_G_ values are mainly due to the larger size of Fe2, which disrupts the confining effect of the hollow cavities in ZIF-8, leading to the introduction of defects during the subsequent pyrolysis process [43,46]. Additionally, the disruption of the carbon matrix by nanoclusters in the target catalysts may also lead to an increase in the degree of defects.

According to previous reports, the active center of these catalysts is typically the Fe site. To further elucidate the chemical state of the Fe center, Fe *K*-edge X-ray absorption fine structure (XAFS) spectroscopy was employed. As illustrated in Fig. 2e, X-ray absorption near-edge structure (XANES) spectroscopy reveals that the edge energies of Fe_DS_-NC and Fe_DS/MC_-NC are located between those of the Fe foil and Fe_2_O_3_. This suggests that the oxidation state of Fe in these samples ranges between 0 and +3. It is noteworthy that Fe_DS/MC_-NC exhibits a slightly lower valence than Fe_DS_-NC, which may be attributed to the presence of zero-valence Fe clusters in Fe_DS/MC_-NC. Additionally, the Fourier-transform (FT) *k*^2^-weighted extended X-ray absorption fine structure (EXAFS) spectra for Fe_DS_-NC show a primary peak ∼1.50 Å and a secondary peak ∼2.20 Å, corresponding to the scattering paths of Fe-N(O) and Fe-Fe, respectively (Fig. 2f) [47,48]. Further, the FT-EXAFS curve-fitting analysis of Fe_DS_-NC was performed in Fig. S19 and Table S2, the Fe-N1 and Fe-N2 exhibit coordination numbers of 2 and bond lengths of 1.90 and 2.08 Å, respectively. Additionally, the Fe-Fe bond features a coordination number of 1 and a bond length of ∼2.52 Å. These observations are consistent with our previous reports. In addition, Fe_DS/MC_-NC displays a new peak at ∼2.60 Å in the EXAFS spectra, corresponding to the scattering path of Fe-O-Fe in Fe_3_O_4_, which further confirms the presence of Fe_3_O_4_ clusters [49]. Notably, the preparation processes for Fe_DS/MC_-NC and Fe_DS_-NC are identical, with the only difference being the Fe2 content. Therefore, there is no difference in the formation mechanism of the dual-Fe pairs and their configuration. EXAFS wavelet transform (WT) analysis further confirms the oxidation state and coordination environment of Fe in Fe_DS/MC_-NC (Fig. 2g–j). The analysis indicates that at low Fe concentrations, Fe is dispersed in pairs, forming an Fe_2_N_6_ configuration with Fe-Fe and Fe-N bonds. As the Fe content increases, Fe or Fe_3_O_4_ clusters are formed near Fe_2_N_6_ sites.

**Figure 3.**
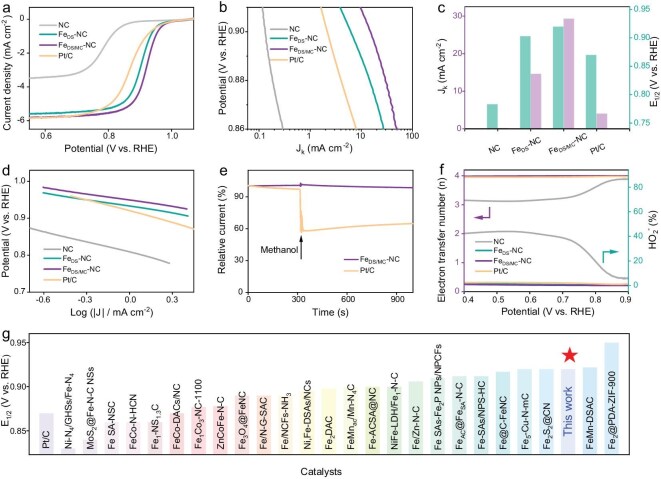
Electrocatalytic performance evaluation. (a) LSV curves. (b) *J*_k_ curves. (c) Performance parameters of *J*_k_ (record at 0.880 V vs RHE) and *E*_1/2_. (d) Tafel slopes. (e) Methanol resistance *i-t* test. (f) Electron transfer number and HO_2_^−^ yield. (g) Comparison of electrocatalytic performances of the Fe_DS/MC_-NC and other recently reported similar materials.

The combination of a porous carbon matrix and Fe_2_N_6_ sites in the target catalysts imparts them with excellent electrocatalytic potential. Thus, the electrocatalytic performance of the as-prepared catalysts toward ORR were conducted in O_2_-saturated 0.1 M KOH using a classical three-electrode system. Cyclic voltammetry (CV) was first performed in O_2_/Ar-saturated KOH solution, and distinct cathodic peaks confirmed the oxygen reduction behavior of the catalysts (Fig. S20). The linear sweep voltammetry (LSV) curves toward different Fe2 additions reveal that the half-wave potentials (*E*_1/2_) initially increase and then decrease (Fig. S21). According to the previous structural analysis, this performance improvement can be attributed to the positive contribution of Fe-based clusters formed near the dual-Fe sites, enhancing the ORR. However, the subsequent decrease in performance may result from the formation of excessive Fe-based clusters, which reduces the density of active sites. The LSV curves at different rotational speeds show a gradient enhancement in current density across all samples (Figs S22, S23), indicating that the electrocatalytic activity of the catalysts is highly dependent on oxygen diffusion. The Koutecký-Levich (K-L) plots derived from the LSV curves at different rotational speeds exhibit a standard linear relationship, confirming first-order reaction kinetics for the catalysts. Notably, as shown in Fig. 3a, Fe_DS/MC_-NC demonstrated an onset potential (*E*_onset_) of 0.990 V versus reversible hydrogen electrode (RHE) and *E*_1/2_ of 0.920 V versus RHE, outperforming Fe_DS_-NC (0.976 and 0.903 V vs RHE), NC (0.874 and 0.783 V vs RHE), and Pt/C (0.983 and 0.870 V vs RHE). Additionally, the electrocatalytic activity of Fe_DS/MC_-NC was comparable to other reported similar catalysts (Fig. 3g and Table S3). Furthermore, Fe_DS/MC_-NC exhibited an impressive kinetic current density of 29.4 mA cm^−2^ at 0.880 V versus RHE (Fig. 3b, c), which was twice as high as Fe_DS_-NC (14.6 mA cm^−2^) and 7.3 times higher than Pt/C (4.0 mA cm^−2^). The favorable kinetics of Fe_DS/MC_-NC were further confirmed by its low Tafel slope value of 56.1 mV dec^−1^, which is superior to Fe_DS_-NC (59.4 mV dec^−1^), NC (93.9 mV dec^−1^), and Pt/C (104.3 mV dec^−1^) (Fig. 3d).

To further investigate the impact of Fe-based atomic clusters on the electrocatalytic performance of Fe_DS/MC_-NC, the catalyst was subjected to acid treatment. The PXRD pattern revealed that the characteristic peaks of Fe-based clusters disappeared after this treatment (Fig. S24a). Subsequent LSV tests showed a significant decrease in both the *E*_1/2_ and limiting current densities of Fe_DS/MC_-NC, corresponding with the loss of the Fe-based clusters (Fig. S24b). These findings further confirmed that the dual-Fe sites and Fe-based clusters work synergistically to drive the efficient ORR electrocatalytic activity of Fe_DS/MC_-NC. Moreover, Fe_DS/MC_-NC demonstrated excellent methanol resistance, showing negligible current disruption after methanol injection into the electrolyte (Fig. 3e). The slight increase in current observed post-injection is mainly due to the temporary rise in methanol concentration near the electrode, as the solubility of oxygen is significantly higher in methanol than in water. In contrast, this level of methanol resistance was not observed in commercial Pt/C, where the current dropped sharply, retaining only ∼60% of its initial value after the same methanol injection. The ORR selectivity of the prepared electrocatalysts was evaluated by LSV testing using a rotating ring disk electrode. As illustrated in Fig. 3f, the electron transfer number (*n*) for Fe_DS/MC_-NC remained consistently close to 4, and the H_2_O_2_ yield was kept below 3% within the voltage range of 0.4–0.9 V versus RHE. In comparison, Fe_DS_-NC and Pt/C showed similar *n* values and H_2_O_2_ yields, while the H_2_O_2_ yield for NC exceeded 40% at 0.4 V versus RHE. These results confirmed that the ORR process in Fe_DS/MC_-NC predominantly followed a highly selective four-electron transfer pathway.

To understand the origin of catalytic activity, the turnover frequency (TOF) values for different catalysts were calculated. As shown in Fig. S25, both Fe_DS_-NC and Fe_DS/MC_-NC exhibit high TOF values, where Fe_DS/MC_-NC is slightly lower than Fe_DS_-NC, which is mainly due to the presence of some less active or even inactive metal clusters. Further, the comparable *C*_dl_ values exhibited by the different catalysts also confirm their similar active surface area (Fig. S26). Therefore, the improvement in Fe_DS/MC_-NC activity is primarily attributed to the intrinsic activity enhancement of dual-Fe sites. The structural characterization of Fe_DS/MC_-NC after a 3000 cycle CV test was carried out. The Raman spectroscopy showed that the D band ∼1350 cm^−1^ exhibited a certain degree of broadening after cycling, which may be attributed to the defects created by the etching of a small amount of carbon during the prolonged cycling process (Fig. S27a). In addition, no significant metal agglomerations were observed in the TEM, HR-TEM, and TEM mapping images of the Fe_DS/MC_-NC electrode after cycling, further demonstrating its well-structured stability (Fig. S27).

**Figure 4.**
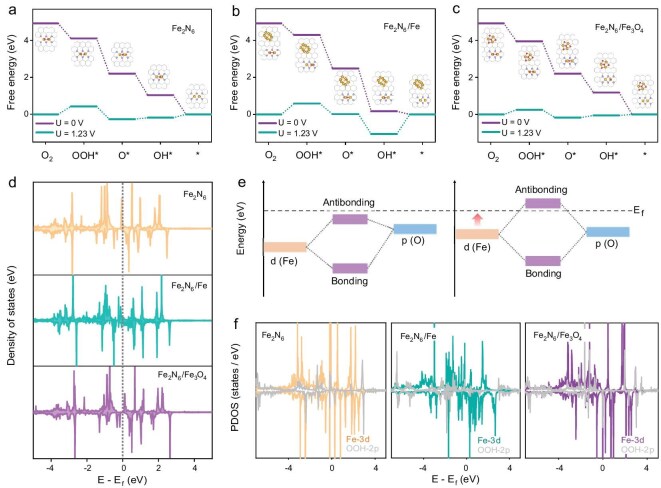
Theoretical calculations. Free energy diagram of the ORR for (a) Fe_2_N_6_, (b) Fe_2_N_6_/Fe, and (c) Fe_2_N_6_/Fe_3_O_4_. (d) DOS of Fe 3*d* for Fe_2_N_6_, Fe_2_N_6_/Fe, and Fe_2_N_6_/Fe_3_O_4_. (e) The possible schematic diagram of bond formation between the catalytic site and the oxygen intermediate. (f) Projected density of states (PDOS) of Fe 3*d* and OOH 2*p* for Fe_2_N_6_, Fe_2_N_6_/Fe, and Fe_2_N_6_/Fe_3_O_4_ after *OOH adsorption at the dual-Fe site.

To gain a deeper understanding of how the composition and spatial structure of active centers influence ORR activity, density-functional theory (DFT) calculations were conducted. According to the analysis of data from HAADF-STEM and XAFS, the electronic effects were explored by introducing Fe and Fe_3_O_4_ clusters near the Fe_2_N_6_ sites to form Fe_2_N_6_/Fe and Fe_2_N_6_/Fe_3_O_4_ models, respectively (Fig. S28). The oxygen adsorption configurations and adsorption energies on the different environmentally active centers were then investigated. As illustrated in Fig. S29 and Table S4, the adsorption configuration of oxygen consistently retained its original bridge adsorption, regardless of whether Fe or Fe_3_O_4_ clusters were introduced near the Fe_2_N_6_ sites. Given that the catalytic center mainly follows a four-electron transfer pathway, free energy diagrams for three types of models were calculated [28]. As illustrated in Fig. 4a–c, the Fe_2_N_6_/Fe_3_O_4_ configuration exhibits optimal ORR catalytic activity, with the RDS being the formation of OOH*. In contrast, the Fe_2_N_6_/Fe model shows a higher theoretical overpotential compared to Fe_2_N_6_, with the RDS shifting from OOH* formation to OH* desorption. This suggests that Fe_3_O_4_ clusters play a positive role in enhancing the catalytic activity of Fe_2_N_6_, while Fe clusters cause over-adsorption of OH* at the Fe_2_N_6_ sites, disrupting the balance between adsorption and desorption of various oxygen-containing intermediates. As a comparison, a theoretical model for the coexistence of Fe and Fe_3_O_4_ clusters in the neighborhood of a dual-Fe site was constructed (Fig. S30a). The Gibbs free energy diagrams show that this catalytic model exhibits a theoretical overpotential of ∼0.32 V, higher than Fe_2_N_6_/Fe_3_O_4_ (0.22 V) but lower than that of Fe_2_N_6_ (0.42 V) (Figs S30b and S31). This suggests that the coexistence of two metal clusters can also significantly improve the electrocatalytic activity. The synergistic mechanism of Fe_3_O_4_ clusters with single-atom Fe sites was also analyzed theoretically. The Gibbs free energy diagrams show that the theoretical overpotential rises from 0.70 V to 1.53 V when Fe_3_O_4_ clusters are located near the Fe sites (Figs S32, S33). This suggests that the introduction of Fe_3_O_4_ clusters near the single-atom Fe sites produces a significant negative effect, which further exacerbates the over-adsorption state of OH* on the active sites, thus increasing the reaction energy barrier.

Furthermore, differential charge density analysis was used to reveal the electron redistribution behavior of Fe-based clusters when positioned near Fe_2_N_6_. As shown in Fig. S34, the combination of Fe_2_N_6_ with Fe clusters resulted in negligible changes in the electronic structure. However, the integration of Fe_3_O_4_ clusters adjacent to Fe_2_N_6_ caused a significant redistribution of the electronic structure at the Fe_2_N_6_ active center. Additionally, the charge density differences of Fe_3_O_4_ loaded on graphene showed no detectable electron transfer, indicating a lack of significant interaction between Fe_3_O_4_ and graphene. This contrast highlights the specific influence of Fe_3_O_4_ clusters on the dual-Fe sites. Notably, the *d*-orbitals of Fe in Fe_3_O_4_ and Fe_2_N_6_ show a certain degree of overlap, further indicating that there is a weak orbital interaction between them (Fig. S35). Meanwhile, the PDOS reveals that the introduction of Fe_3_O_4_ clusters leads to a significant change in the electronic structure of the dual-Fe sites (Fig. 4d). Specifically, with the addition of Fe_3_O_4_, the orbitals near the Fermi level in the dual-Fe site shift to the right, and the orbital with spin-down moves toward the Fermi level. When the reaction intermediate adsorbs, the spin-down orbitals are more likely to interact with the electronic orbitals of the reaction intermediate. This interaction leads to the formation of antibonding orbitals above the Fermi energy level and electron-occupied bonding orbitals below the Fermi energy level, thus enhancing the adsorption of the reaction intermediates and ultimately lowering the reaction energy barrier (Fig. 4e) [23,50]. To provide a clearer understanding of the spin polarization, the *d*-band centers of different model catalysts were calculated based on the density of states analysis. As shown in Table S5, there is minimal deviation between the spin-up and spin-down *d*-band centers for the dual-Fe sites in the Fe_2_N_6_ and Fe_2_N_6_/Fe structures. In contrast, in the Fe_2_N_6_/Fe_3_O_4_ model, the spin-up *d*-band center is at −1.978 eV, while the spin-down *d*-band center is at −0.955 eV. The significant rightward shift of the spin-down *d*-band center in Fe_2_N_6_/Fe_3_O_4_ is favorable for the formation of reaction intermediates [14,49].

Combined with Gibbs free energy and PDOS analyses, it can be demonstrated that the presence of Fe_3_O_4_ clusters significantly reduces the theoretical overpotential of dual-Fe sites, primarily manifested in the optimization of the adsorption and desorption processes of the key reaction intermediate OOH*. Therefore, the PDOS analysis of OOH* adsorption at the active site reveals that, unlike in Fe_2_N_6_ and Fe_2_N_6_/Fe, the spin-down orbitals of the Fe site in Fe_2_N_6_/Fe_3_O_4_, located near the Fermi energy level, interact with the electronic orbitals of OOH* (Fig. 4f). This interaction can induce electron-occupied bonding orbitals and electron-unoccupied antibonding orbitals, which enhances the adsorption of OOH* intermediates. This finding aligns with the conclusions drawn from the PDOS of the initial structure. Additionally, differential charge density calculations for OOH* intermediates after adsorption on the active site reveal significant electron interactions involving the Fe_3_O_4_ clusters (Fig. S36). This indicates that Fe_3_O_4_ clusters play a role in the adsorption process of the OOH* intermediates.

**Figure 5.**
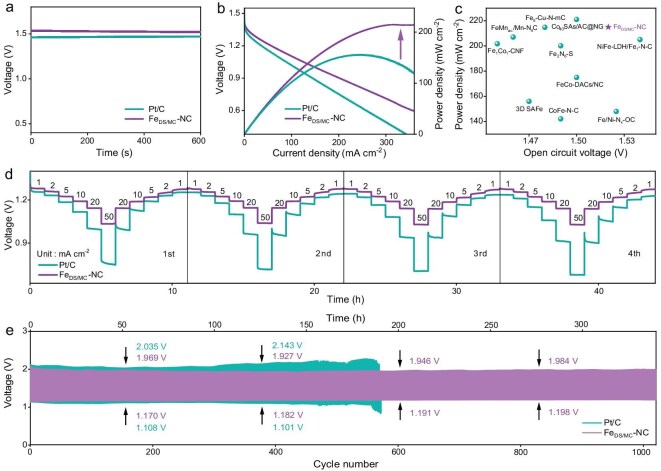
Performance of Zn-air batteries based on Fe_DS/MC_-NC and Pt/C. (a) Open-circuit voltage. (b) Discharge polarization and power density curves. (c) Comparison of zinc-air battery performances of the Fe_DS/MC_-NC and other recently reported similar materials. (d) Rate-performance. (e) Discharge/recharge cycling curves at the current density of 10 mA cm^−2^.

Encouraged by the excellent ORR performance of Fe_DS/MC_-NC, it was then employed as an air cathode in a home-made aqueous zinc-air battery (ZAB) to assess its applicability in energy conversion systems. A polished zinc plate was used as the metal anode, with a 6.0 M KOH solution containing 0.2 M Zn(CH_3_COO)_2_ as the electrolyte. Commercial Pt/C served as the control sample for the air cathode. As illustrated in Fig. 5a, the Fe_DS/MC_-NC-based ZAB achieved an open-circuit voltage (OCV) of up to 1.52 V, significantly higher than the commercial Pt/C with an OCV of 1.47 V. Meanwhile, based on the discharge curve, the ZAB constructed with Fe_DS/MC_-NC delivers a peak power density of 214.6 mW cm^−2^ at a current density of 317 mA cm^−2^, outperforming the commercial Pt/C, which has a peak power density of 155.4 mW cm^−2^ at 244 mA cm^−2^ (Fig. 5b). Additionally, the Fe_DS/MC_-NC-driven ZAB performance shows a significant advantage over other reported electrocatalysts of the same type (Fig. 5c and Table S6). Rate performance is also considered a crucial factor in evaluating battery performance. As shown in Fig. 5d, the Fe_DS/MC_-NC assembled ZAB still maintains a highly stable voltage balance under continuous and repeated gradient current densities, demonstrating its flexibility in responding to energy output in various states. In contrast, the Pt/C assembled ZAB shows poor rate performance, particularly at high current densities, with a more pronounced difference compared with Fe_DS/MC_-NC. Constant current charge/discharge tests reveal that Fe_DS/MC_-NC maintains a relatively narrow voltage window after >1000 cycles (Fig. 5e). Notably, the discharge process, where the ORR occurs, displays an exceptionally stable voltage plateau, further confirming the excellent cycling durability of Fe_DS/MC_-NC. To bring the target catalyst closer to practical applications, an all-solid-state ZAB was developed using Fe_DS/MC_-NC as the air cathode and a zinc plate as the anode [51]. As illustrated in Fig. S37, the single cell exhibits an OCV as high as 1.47 V. Additionally, it achieves a peak power density of 69.1 mW cm^−2^ at a current density of 101 mA cm^−2^. Furthermore, the cycling charge/discharge capability for the all-solid-state battery assembled using Fe_DS/MC_-NC was further explored at 2.0 mA cm^−2^. After continuous discharge and charge tests for 20 h, the voltage gap of Fe_DS/MC_-NC was almost constant, proving its favorable cycling performance (Fig. S38).

## CONCLUSIONS

In summary, we successfully positioned Fe-based atomic clusters adjacent to dual-Fe sites to enhance the ORR performance of DMCs. The co-existence of dual-Fe sites and Fe-based atomic clusters was achieved through a straightforward host-guest capping and one-step heat treatment strategy. Precise control of Fe doping in the pyrolysis precursor allowed for bidirectional regulation of the density of dual-Fe sites and Fe-based atomic clusters, which is essential for the electronic structure of dual-Fe sites. Electrochemical tests demonstrated a significant improvement in ORR performance with the presence of atomic clusters. When used as a catalyst for the air electrode in ZAB, it achieves a maximum power density of 214.6 mW cm^−2^ at a current density of 317 mA cm^−2^, with minimal expansion of the voltage window even after >1000 charge-discharge cycles. Theoretical calculations indicate that the presence of Fe-based atomic clusters maintains the bridging adsorption pattern of oxygen at the dual-Fe sites. Additionally, Fe_3_O_4_ clusters significantly enhance the ORR performance of the dual-Fe sites by inducing spin polarization in the electronic structure of the active sites. The spin-down orbitals of the active sites, located near the Fermi energy level, interact with the electronic orbitals of OOH*, thereby accelerating the reaction kinetics. This work paves the way for a new perception of the structure design and performance enhancement of DMCs.

## Supplementary Material

nwaf356_Supplemental_File
