# Supplementary material for: Installation of metal clusters adjacent to dual-Fe sites for enhanced oxygen reduction
Source: Natl Sci Rev. 2025 Aug 28;12(10):nwaf356. doi: 10.1093/nsr/nwaf356 (PMC12485987; doi:10.1093/nsr/nwaf356)
Supplement: nwaf356_Supplemental_File [file nwaf356_supplemental_file.pdf]

Supporting Information

Installation of metal clusters adjacent to dual-Fe sites for enhanced oxygen reduction

Ming Liu, Xuemin Wang, Shoufu Cao, Xiaoqing Lu, Wei Li, Na Li and Xian-He Bu**

Experimental section

Preparation of ZIF-8: $Zn(NO_3)_2$ $6H_2O$ (4 mmol) dissolved in 20 mL oftai methanol was added in 40 mL methanolic solution containing 2-methylimidazole (15 mmol) and triethylamine (20 μL), then stirred vigorously for 10 h at room temperature. The white precipitate was collected by centrifugation and washed several times with methanol and dried at 60 ℃ overnight.

Preparation of ZIF-8@Fe2-x (x = 5, 10, 15, 20, 40, and 80): The samples were synthesized according to our previous method with minor modifications.[1] Specifically, $Zn(NO_3)_2$ $6H_2O$ (4 mmol) was added in 20 mL methanol and stirred for 20 min to form solution A; 2-methylimidazole (15 mmol) and triethylamine (20 μL) were added in 20 mL methanol and stirred for 20 min to form solution B. Simultaneously, x mg Fe2 (cyclopentadienyliron dicarbonyl dimers) (x = 5, 10, 15, 20, 40, and 80) was dissolved in 20 mL methanol and stirred for 30 min and then transferred to the solution B for stirred another 5 min. The mixed solution B was then added to solution A and stirred continuously for 10 h at room temperature away from light. The resulting light yellow precipitate was obtained by centrifugation and washed with methanol several times and finally dried at 60 ℃ overnight.

Preparation of NC: The 200 mg of ZIF-8 was transferred into a ceramic crucible and placed in a tube furnace. Then the sample was heated to 950 ℃ (4 ℃ min^{-1}) for 3 h under a flowing Ar atmosphere. The as-prepared black products were directly used without any post-treatment and named NC.

Preparation of Fe-NC-x: The 200 mg of ZIF-8@Fe2-x was transferred into a ceramic crucible and placed in a tube furnace. Then the sample was heated to 950 ℃ (4 ℃ min^{-1}) for 3 h under a flowing Ar atmosphere and then naturally cooled to room temperature. The as-prepared black products were directly used without any post-treatment and named Fe-NC-x (x = 5, 10, 15, 20, 40, and 80). Except for special instructions, Fe-NC-15 and Fe-NC-40 are respectively represented as Fe_{DS}-NC and $Fe_{DS/MC}$-NC.

Acid treatment of $Fe_{DS/MC}$-NC: The black powder of $Fe_{DS/MC}$-NC was added to 2.0 M H_2SO_4 and then stirred at 70 ℃ for 10 h, followed by washing several times with deionized water, and finally dried at 60 ℃ overnight.

Material Characterization

The Powder X-ray diffraction (PXRD) data was recorded on the Rigaku Smart Lab X-ray diffractometer to analyze the structure of the samples. The field emission scanning electronic microscope (FE-SEM) and transmission electron microscope (TEM) images were achieved on JSM-7800F and JEM-2800 to determine the morphologies and sizes of the samples, respectively. High-resolution TEM (HR-TEM), scanning transmission electron microscope (STEM), and energy dispersive spectroscopy (EDS) mapping images were also obtained using JEM-2800 operating at an accelerating voltage of 200 kV. Aberration-corrected high-angle annular dark-field scanning transmission electron microscopy (HAADF-STEM) was performed on Thermo TiTAN, Themis Z. The X-ray photoelectron spectroscopy (XPS) was performed on a Thermo Scientific ESCALAB 250Xi. Raman measurements were recorded on a JMS1000 (Edinburgh instruments) with a 532 nm laser excitation. Nitrogen adsorption isotherms were obtained on ASAP 2460 (Micromeritics) at 77 K, and the surface area was calculated by the Brunauer-Emmett-Teller (BET) method. The inductively coupled plasma mass spectrometry (ICP-MS) was achieved on an Agilent 7800 to analyze the content of Fe. Fourier transform infrared (FT-IR) spectra of the samples were performed with an FT-IR spectrometer (TENSOR 37).

X-ray Adsorption Spectrum

The Fe *K*-edge analysis was carried out with Si (111) crystal monochromators on the BL11B beamlines at the Shanghai Synchrotron Radiation Facility (SSRF) (Shanghai, China). Prior to the beamline analysis, samples were pressed into thin sheets with 1 cm in diameter and sealed using Kapton tape film. The extended X-ray absorption fine structure (EXAFS) spectra were performed at room temperature using a 4-channel Silicon Drift Detector (SDD) Bruker 5040. Fe *K*-edge EXAFS spectra were recorded in transmission mode. Negligible changes in the line shape and peak position

of Fe *K*-edge XANES spectra were observed between two scans taken for a specific sample. The EXAFS spectra of these standard samples (Fe foil, Fe_2O_3, and Fe_3O_4) were recorded by transmission mode. The spectra were processed and analyzed by the software codes Athena and Artemis.

Electrochemical measurements

All electrochemical measurements were carried out with a Chenhua electrochemical workstation (CHI-760E) equipped with a standard three-electrode system. The Ag/AgCl electrode (1.0 M KCl) and the carbon rod were used as the reference electrode and the counter electrode, respectively. The polished glassy carbon rotating disk electrode (RDE, 5 mm in diameter) and rotating ring disk electrode (RRDE, 4 mm in diameter) were used as working electrodes. Catalyst inks were prepared by mixing 2 mg of catalyst into a mixture of 300 μL of deionized water, 90 μL of isopropanol, and 10 μL of Nafion solution (5% by weight) and ultrasonically dispersed for 30 min. The catalyst loading was 0.26 mg cm^{-1}. The linear sweep voltammetry (LSV) curves were recorded in an O_2-saturated 0.1 M KOH aqueous solution with different rotation speeds at a scan rate of 10 mV s^{-1}. All LSV curves were obtained with iR-corrected. Cyclic voltammograms (CV) measurements were recorded with a scan rate of 20 mV s^{-1} in the Ar or O_2-saturated 0.1 M KOH solution.

The dynamic characteristics of the catalyst were calculated by the Koutechy-Levich (K-L) equation:

$$\frac{1}{J} = \frac{1}{J_L} + \frac{1}{J_K} \qquad (1)$$

where J, J_L, and J_K are the measured current density, diffusion limiting current density, and kinetic current density, respectively.

The electron transfer number (n) and yields of the peroxide species ($\%HO_2^-$) were obtained from RRDE tests via the following equations:

$$\mathrm{n} = 4 \times \frac{I_d}{I_r/N + I_d} \qquad (2)$$

$$\%HO_2^- = 200 \times \frac{I_r/N}{I_r/N + I_d} \qquad (3)$$

where I_d and I_r represent the disk current and ring current, respectively. The N represents the current collection efficiency of the RRDE, which was determined to be 0.37.

The turnover frequency (TOF) values of the samples were calculated from the mole number of active Fe sites via the following equation:

$$TOF = \frac{j \times A}{4 \times m \times F} \quad (4)$$

where J and A stand for the measured current density and surface area of the electrode, respectively. 4 is the number of electrons transferred during oxygen reduction. The m represents the mole number of active sites, and F represents the Faraday constant (F = 96485 C mol^{-1}).

Aqueous Zn-air battery assembly

The liquid Zn-air battery was assembled and tested using a homemade two-electrode electrochemical setup. The catalyst inks were loaded on the carbon paper (1 mg cm^{-2}) and assembled with a gas diffusion layer used as the cathode. The polished zinc foil is used as anode. A mixed solution containing 6.0 M KOH and 0.2 M zinc acetate was used as an electrolyte. The linear sweep voltammetry (LSV) curves were recorded on CHI-760E at a scan rate of 5 mV s^{-1}. The cycling tests were carried out on a LAND CT2001A instrument at a current density of 10 mA cm^{-2}. As a control, the commercial Pt/C was also assembled in a similar pattern.

The all-solid-state Zn-air battery assembly

The gel polymer electrolyte was obtained by the following process: 6.3 g potassium hydroxide, 0.2 g zinc oxide, 1.0 g acrylic acid, and 0.15 g N,N'-methylene-bis(acrylamide) were successively added to 10 mL deionized water and stirred for 5 min. Then, the light-yellow solution was obtained by filtering off the insoluble white precipitate. Next, 75 μL of 0.3 M potassium persulfate was added to the clear solution under strong agitation. After about 3-5 seconds, the solution was quickly poured onto a smooth surface plate and allowed to stand for one hour to obtain the gel polymer electrolyte with a thickness of about 2 mm. To assemble the all-solid-state Zn-air

battery, the polished zinc foil and carbon cloth loaded with catalyst were placed on both sides of the gel polymer electrolyte to form a sandwich-like micro flexible device.

Computational details

The theoretical calculations refer to our previous work.[1] Specifically, all calculations were carried out using spin-polarized density functional theory (DFT) as implemented in Vienna Ab initio Simulation Package (VASP) 6.1.0[2] with Perdew-Burke-Ernzerhof (PBE)[3] generalized gradient approximation (GGA). The cutoff energy was set as 450 eV after cutoff testing, and the *k*-points were set to be 2 × 2 × 1 and 11 × 11 × 1 for the geometry optimization and density of state calculation, respectively. The van der Waals interactions were considered using the method of the Grimme (DFT + D3). The distance between two layers in the z direction is set to be 15 Å to ignore specular interaction. The electronic energy and forces were converged to within 10^{-5} eV and 0.02 eV/Å, respectively. The effect of water was considered with the VASP implicit solvent model.[4]

Changes of Gibbs free energy were calculated by the computational hydrogen electrode (CHE) model,[5] in which the reaction: H^+ (aq) + e^- = 1/2 H_2 (g) is equilibrated at 0 V *vs*. RHE at all pH values. The change of Gibbs free energy (ΔG) for each elementary step was defined as:[6]

$$\Delta G = \Delta E + \Delta E_{\mathrm{ZPE}} - \mathrm{T}\Delta S + \Delta G_{\mathrm{U}} + \Delta G_{\mathrm{pH}} \qquad (5)$$

where ΔE is the reaction adsorption energy, ΔE_{ZPE} and ΔS refer to the zero-point energy (ZPE) and the entropy difference between the products and the reactants at room temperature (T = 298.15 K), respectively. ΔG_U is the contribution of the applied electrode potential (U) to ΔG, which is set to 0 V. The ΔG_{pH} is the free energy contribution due to the variations in H concentration, and the contribution of pH was excluded from consideration.

The ORR pathway could be outlined in the following elementary steps:

$$O_2 + * (H^+ + e^-) \rightarrow *OOH \qquad (6)$$

$$*OOH + (H^+ + e^-) \rightarrow *O + H_2O \qquad (7)$$

$$*O + (H^+ + e^-) \rightarrow *OH \qquad (8)$$

$$*OH + (H^+ + e^-) \rightarrow H_2O + * \qquad (9)$$

The ORR activity was judged via the overpotential, which is defined as:

$$\eta_{ORR} = \max\,[G_{(1)}, G_{(2)}, G_{(3)}, G_{(4)}] + 1.23\text{ eV} \qquad (10)$$

where max denotes the maximum of the four values in the parentheses.

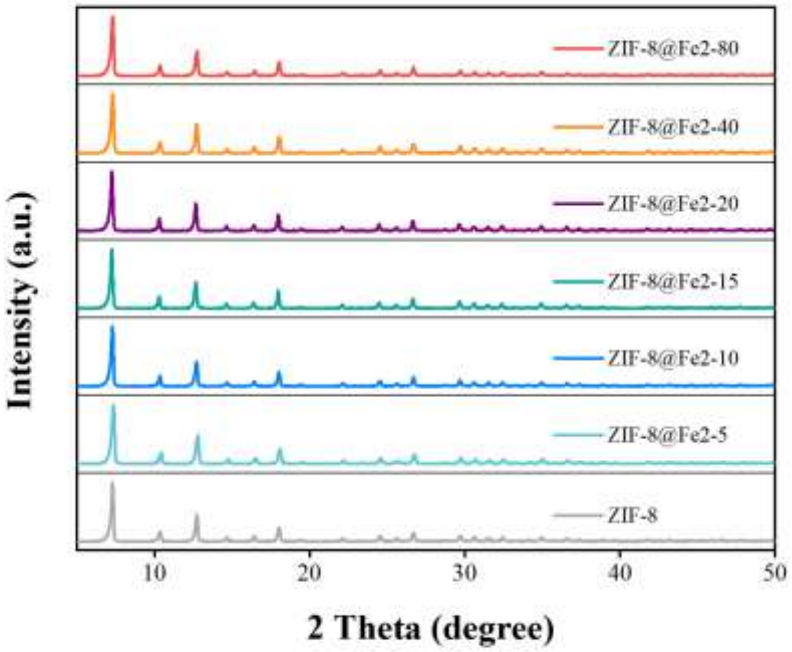

Figure S1. The PXRD pattern of samples.

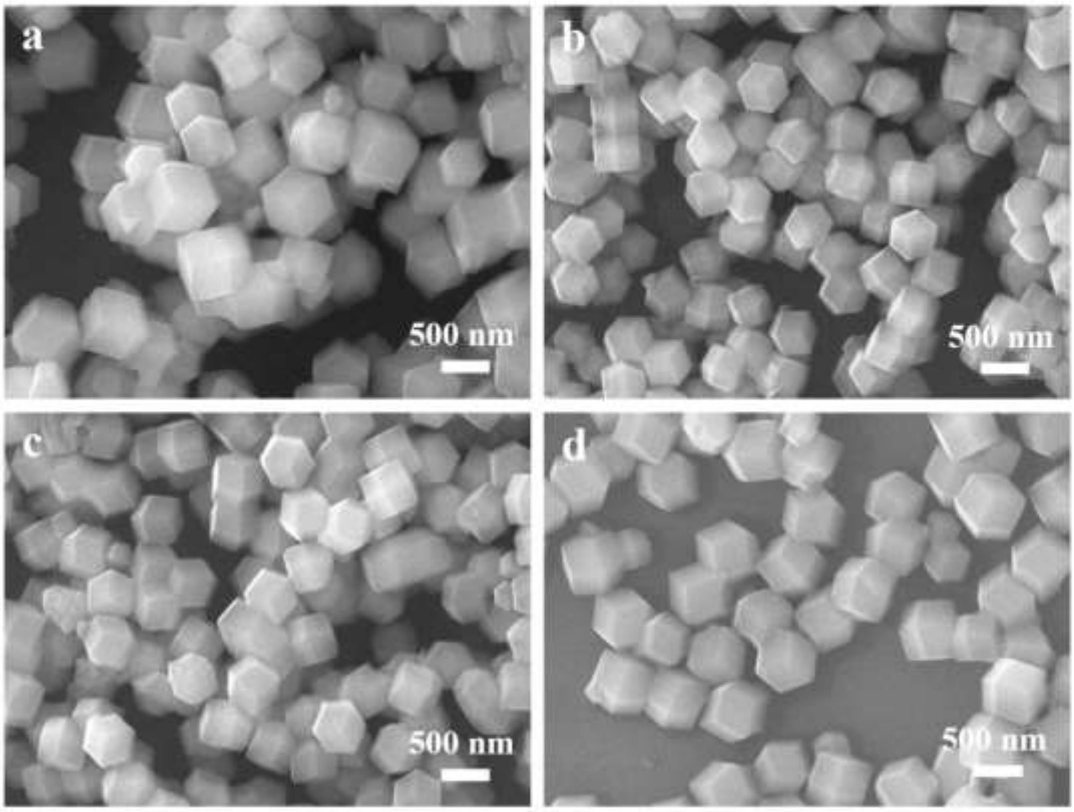

Figure S2. SEM images of a) ZIF-8, b) ZIF-8@Fe2-5, c) ZIF-8@Fe2-10, and d) ZIF-8@Fe2-15.

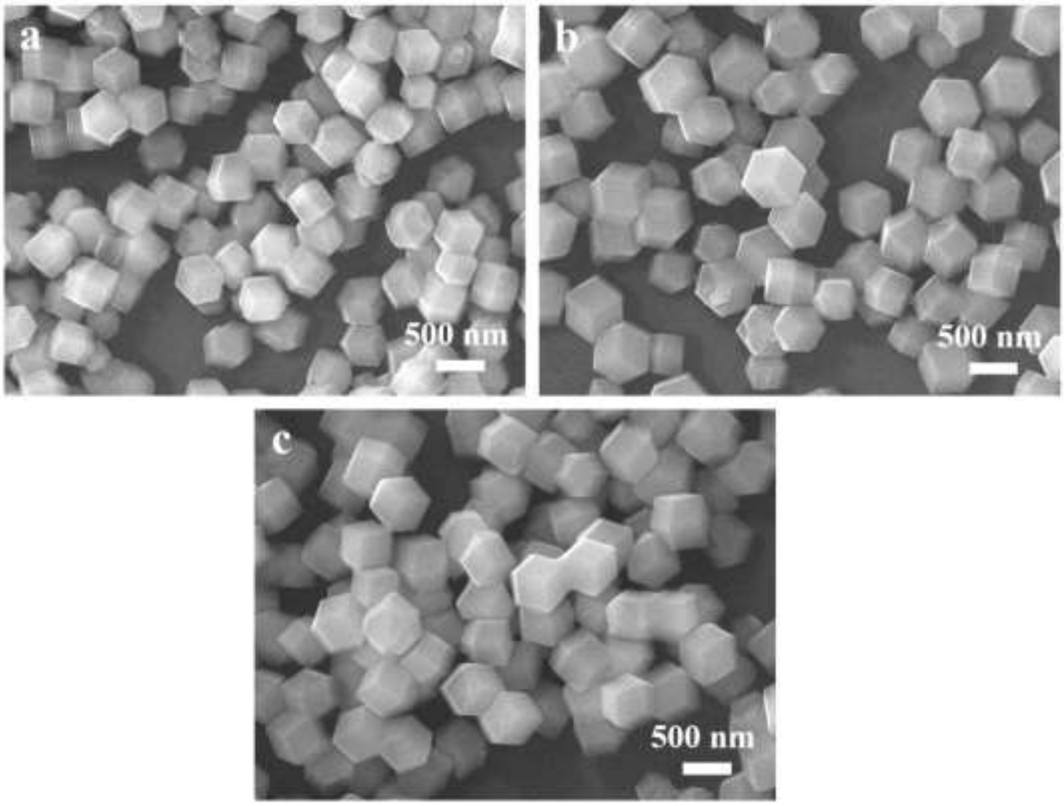

Figure S3. SEM images of a) ZIF-8@Fe2-20, b) ZIF-8@Fe2-40, and c) ZIF-8@Fe2-80.

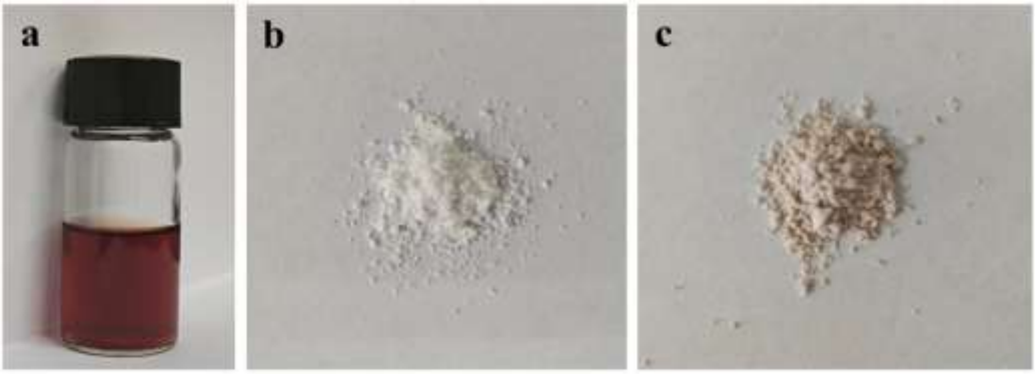

Figure S4. The optical photographs of a) Fe2 dissolved in methanol, b) ZIF-8, and c) ZIF-8@Fe2-40.

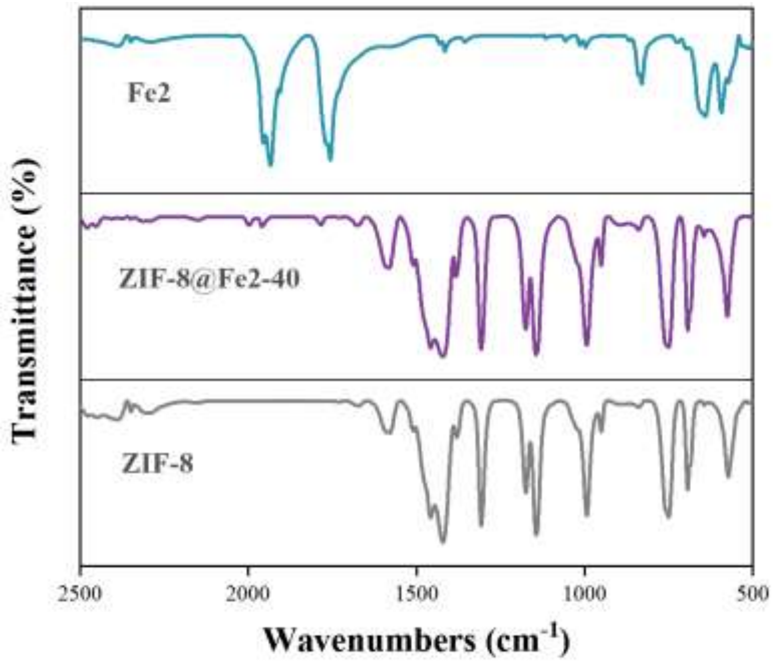

Figure S5. FT-IR spectra of ZIF-8, Fe2, and ZIF-8@Fe2-40.

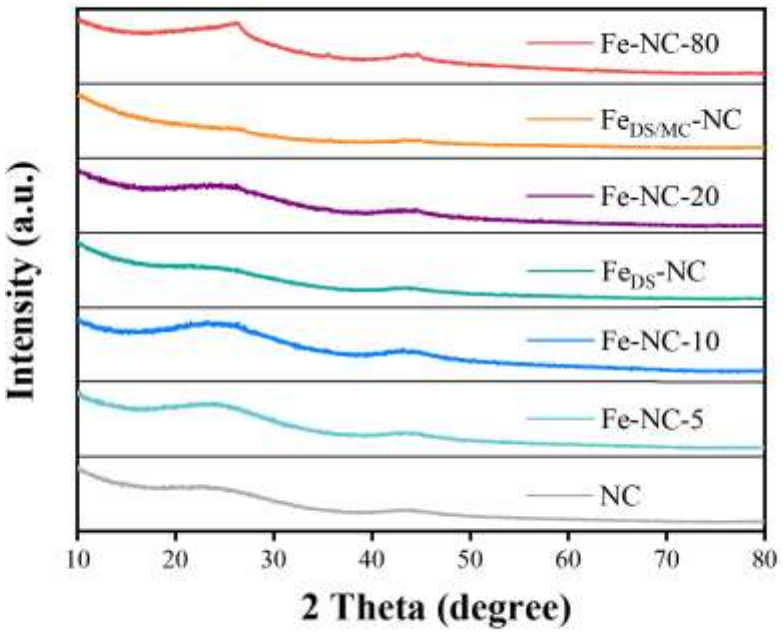

Figure S6. The PXRD patterns of samples.

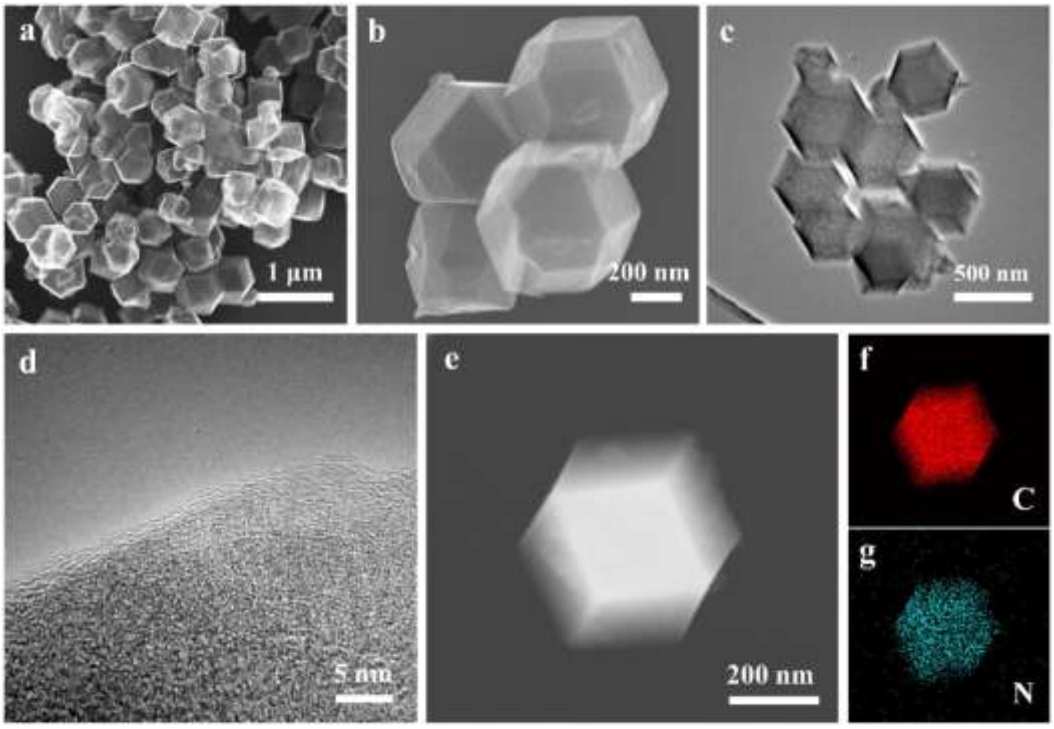

Figure S7. Electron microscopy characterization of NC. a, b) SEM images. c) TEM and d) HR-TEM images. e) STEM and corresponding TEM mapping images for f) C and g) N.

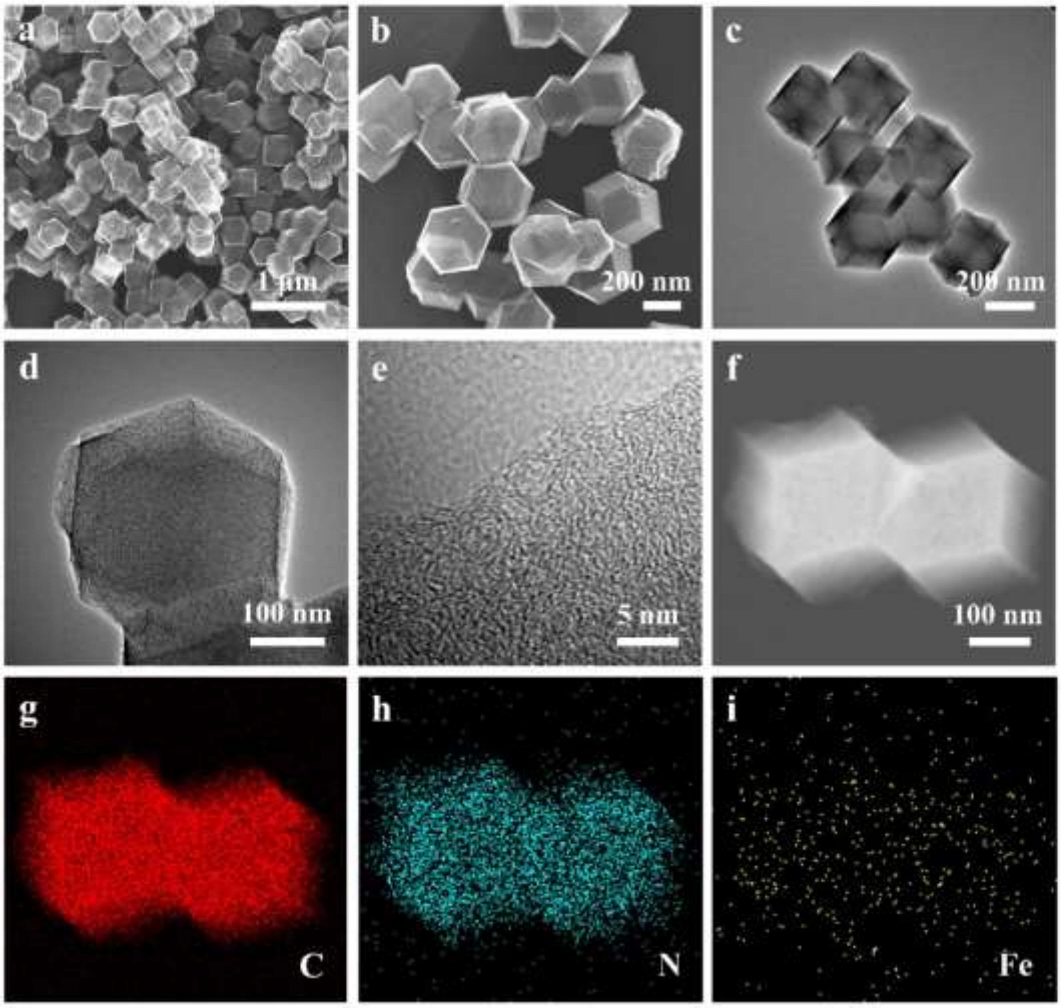

Figure S8. Electron microscopy characterization of Fe-NC-5. a, b) SEM images. c, d) TEM and e) HR-TEM images. f) STEM and corresponding TEM mapping images for g) C, h) N, and i) Fe.

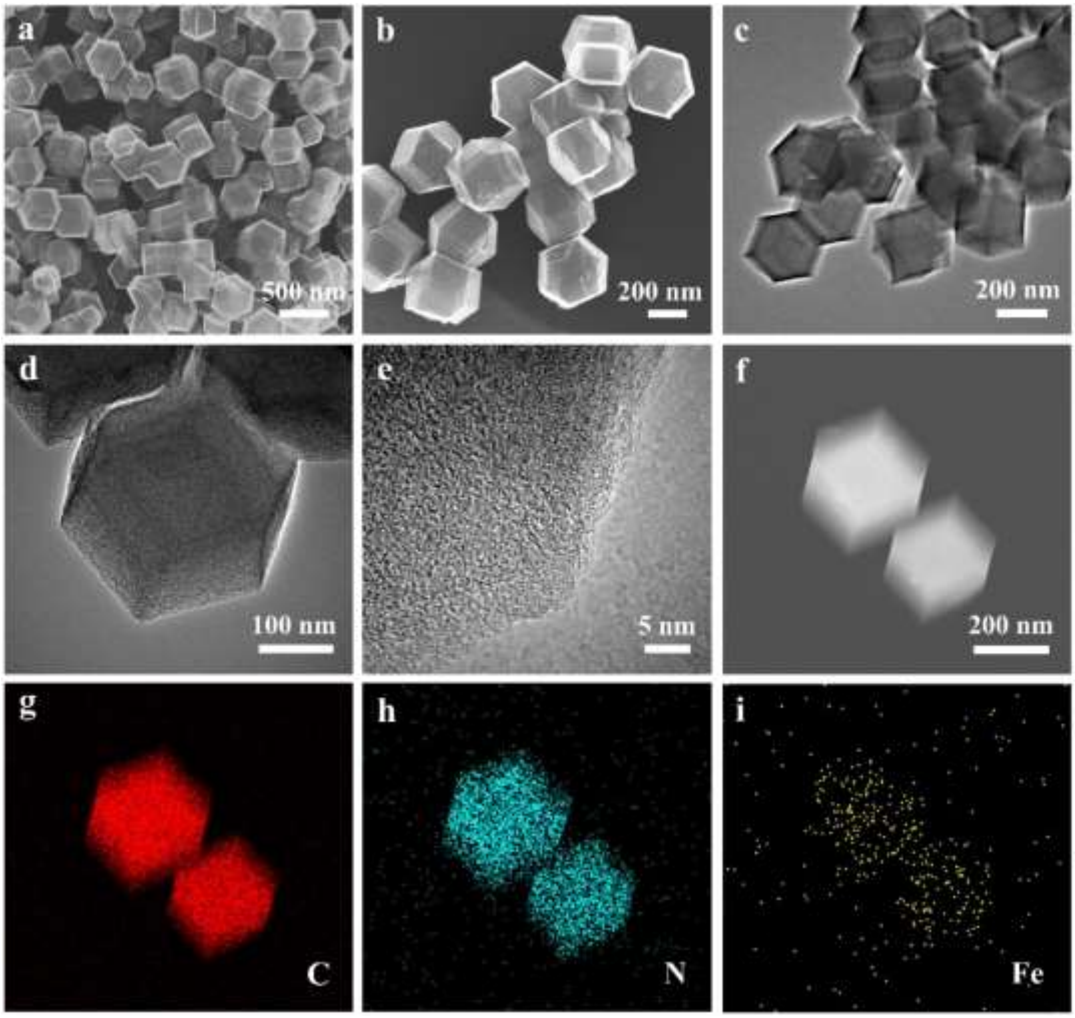

Figure S9. Electron microscopy characterization of Fe-NC-10. a, b) SEM images. c, d) TEM and e) HR-TEM images. f) STEM and corresponding TEM mapping images for g) C, h) N, and i) Fe.

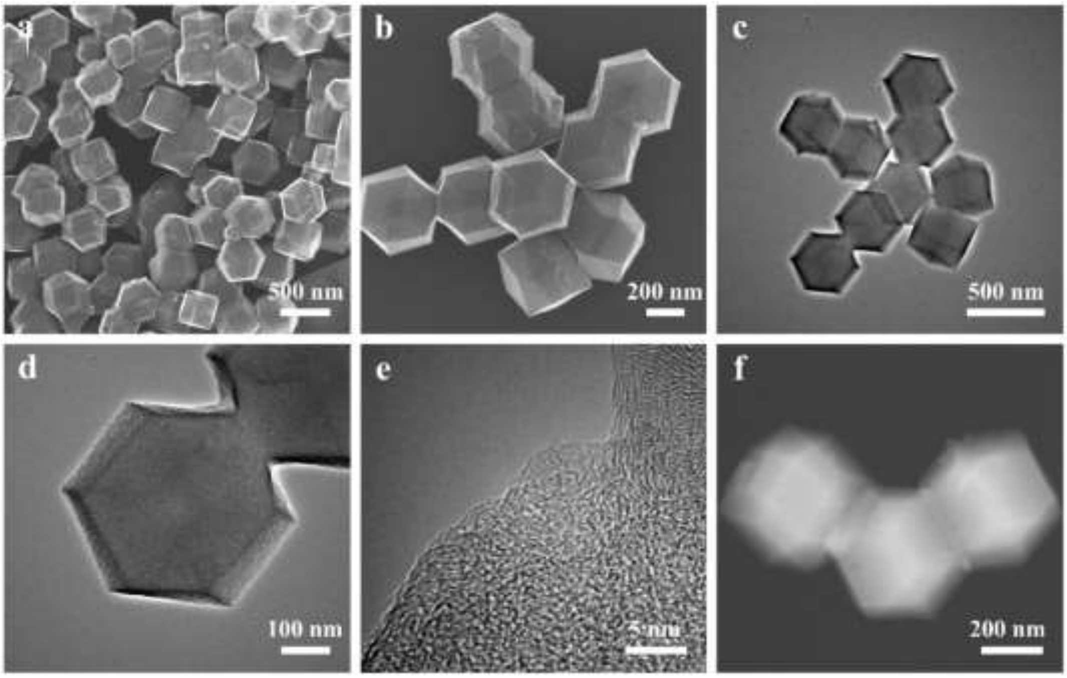

Figure S10. Electron microscopy characterization of Fe_{DS}-NC. a, b) SEM images. c, d) TEM and e) HR-TEM images. f) STEM image.

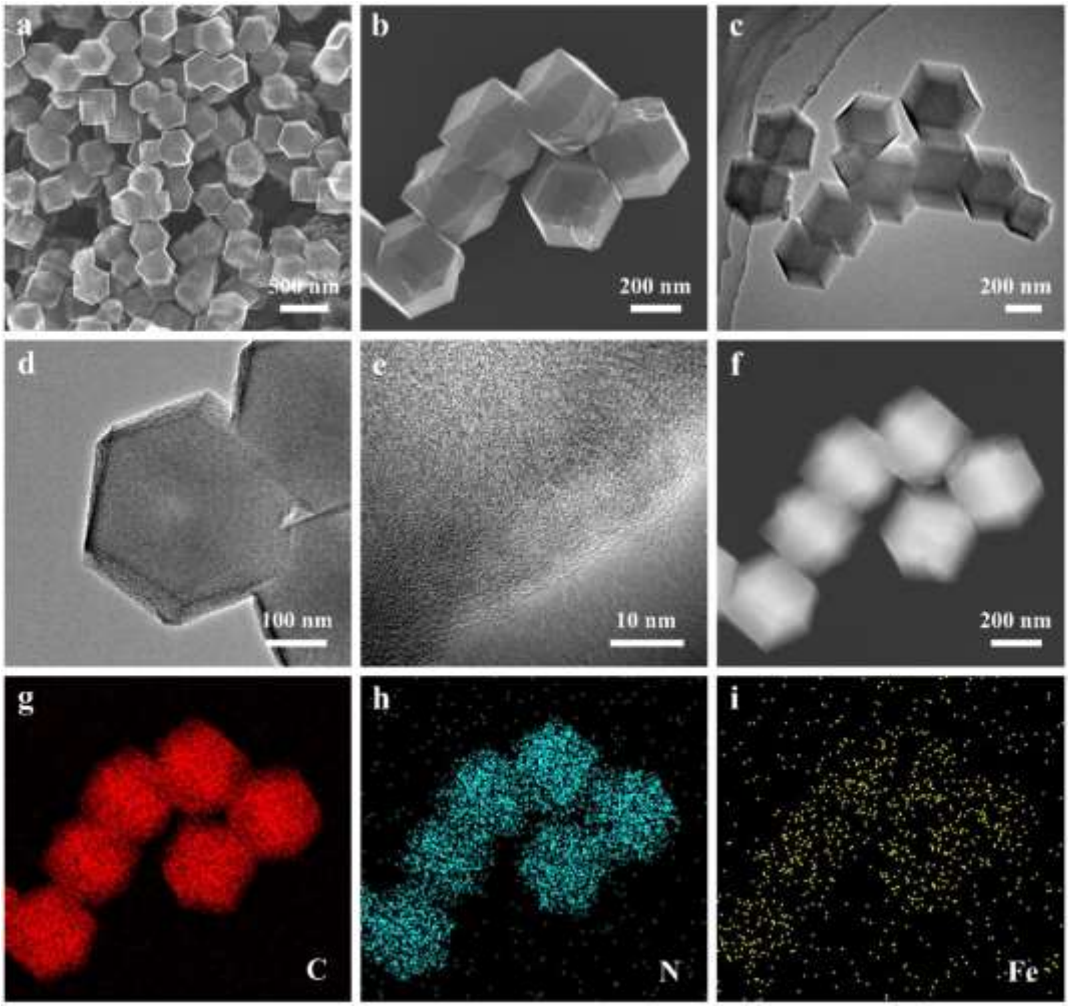

Figure S11. Electron microscopy characterization of Fe-NC-20. a, b) SEM images. c, d) TEM and e) HR-TEM images. f) STEM and corresponding TEM mapping images for g) C, h) N, and i) Fe.

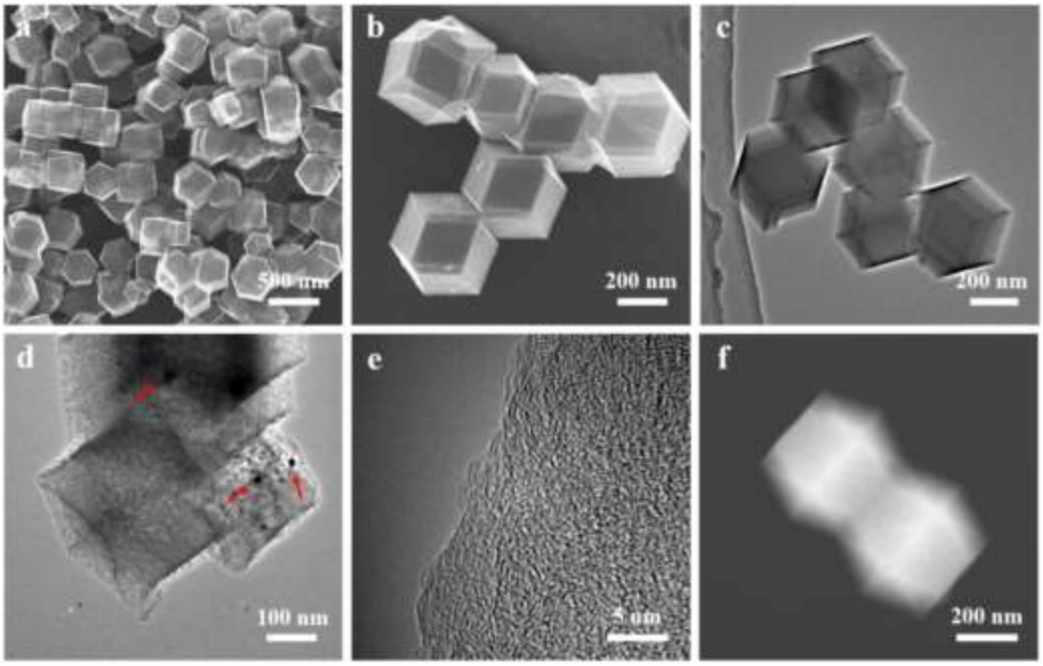

Figure S12. Electron microscopy characterization of $Fe_{DS/MC}$-NC. a, b) SEM images. c, d) TEM and e) HR-TEM images. f) STEM image.

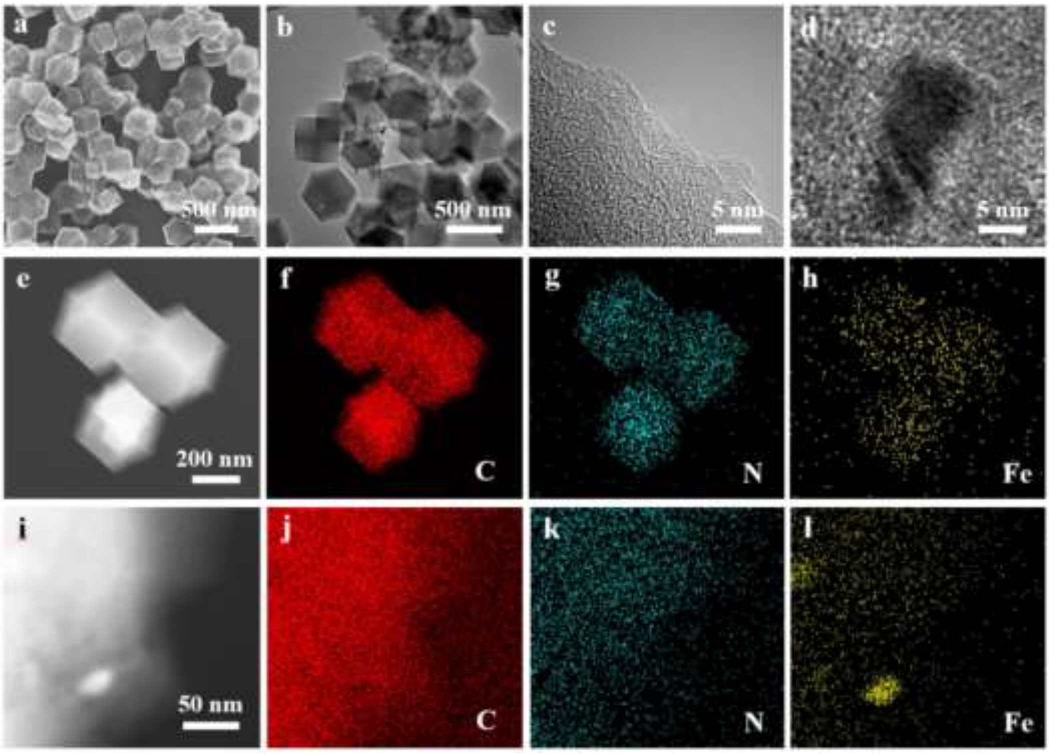

Figure S13. Electron microscopy characterization of Fe-NC-80. a) SEM images. b) TEM and c, d) HR-TEM images. e, i) STEM and corresponding TEM mapping images for f, j) C, g, k) N, and h, l) Fe.

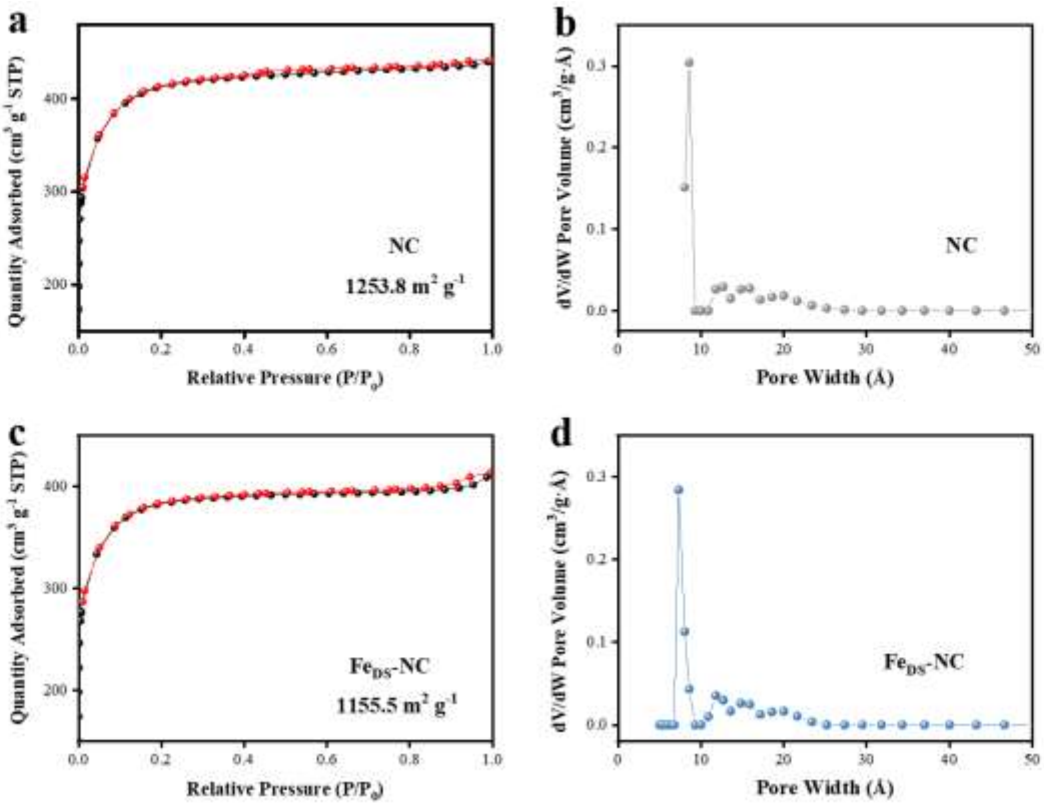

Figure S14. a, c) The nitrogen adsorption-desorption isotherms and b, d) pore size distributions for NC and Fe_{DS}-NC, respectively.

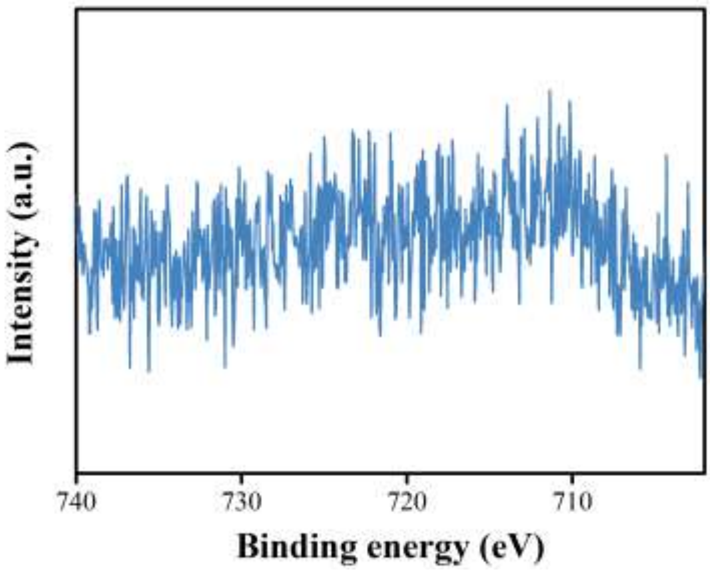

Figure S15. High-resolution Fe 2*p* XPS spectrum of Fe_{DS}-NC.

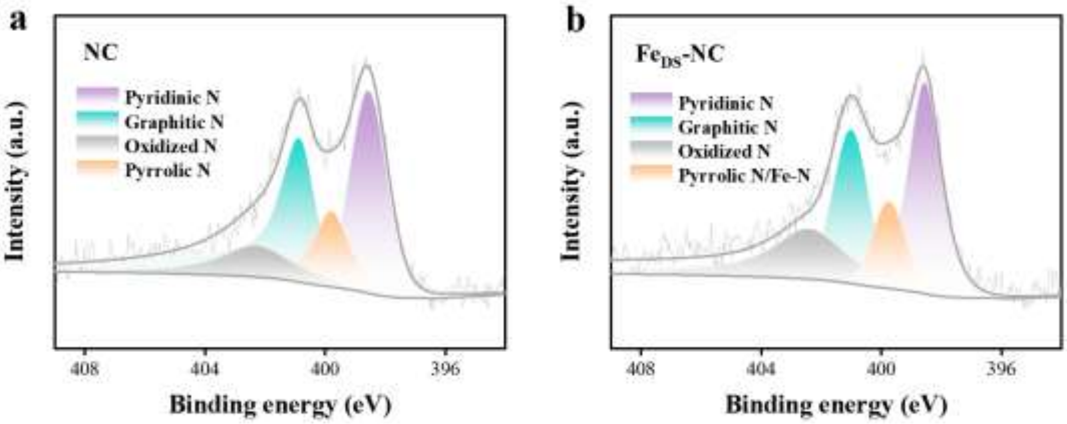

Figure S16. High-resolution N 1*s* XPS spectra of a) NC and b) Fe$_{DS}$-NC.

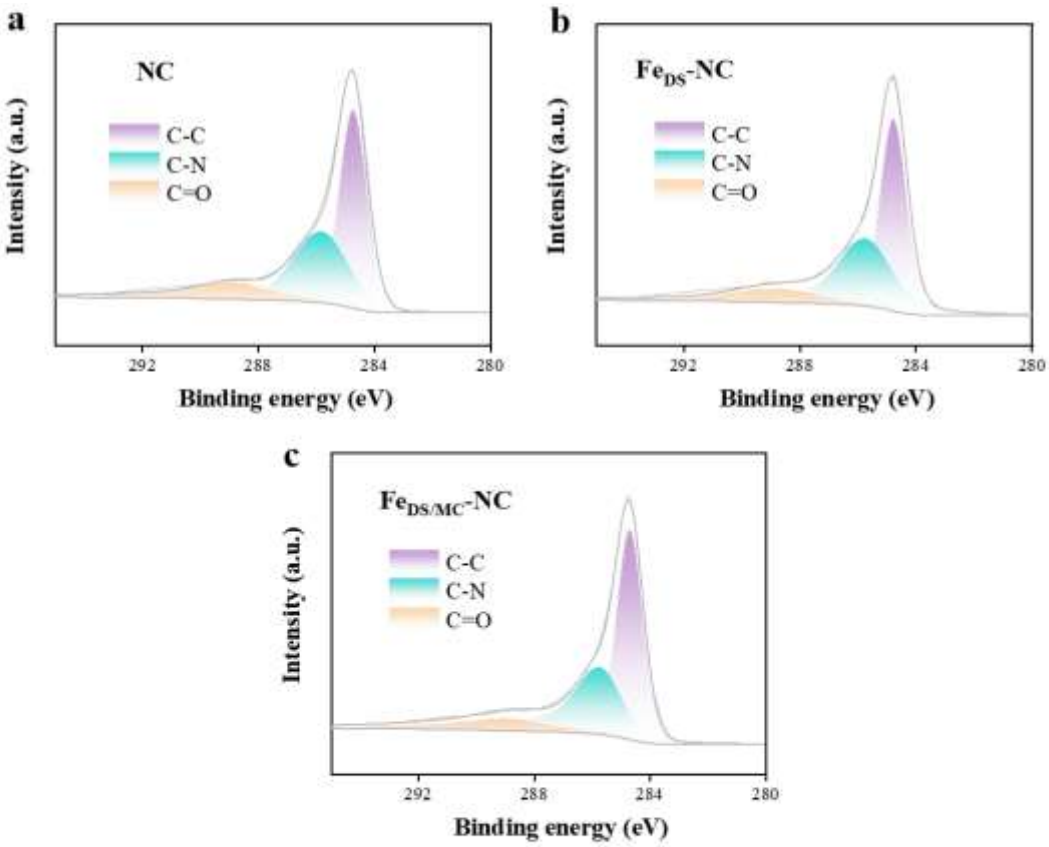

Figure S17. High-resolution C 1*s* XPS spectra of a) NC, b) Fe$_{DS}$-NC, and c) Fe$_{DS/MC}$-NC.

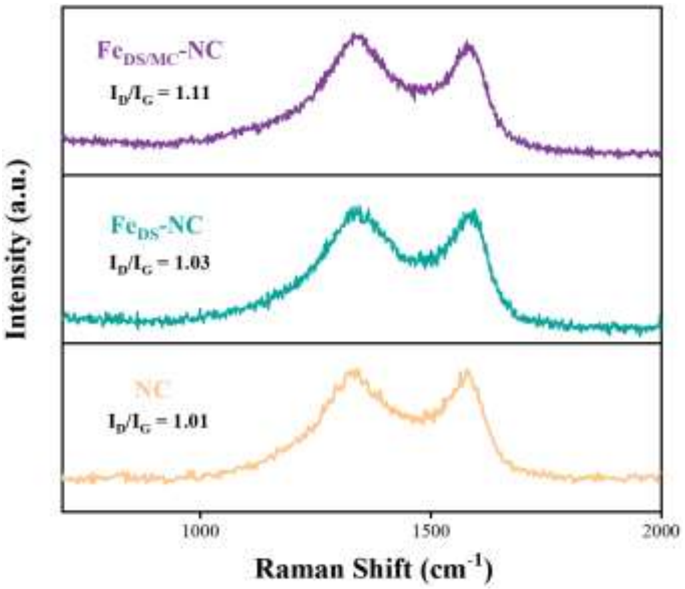

Figure S18. Raman spectra of samples.

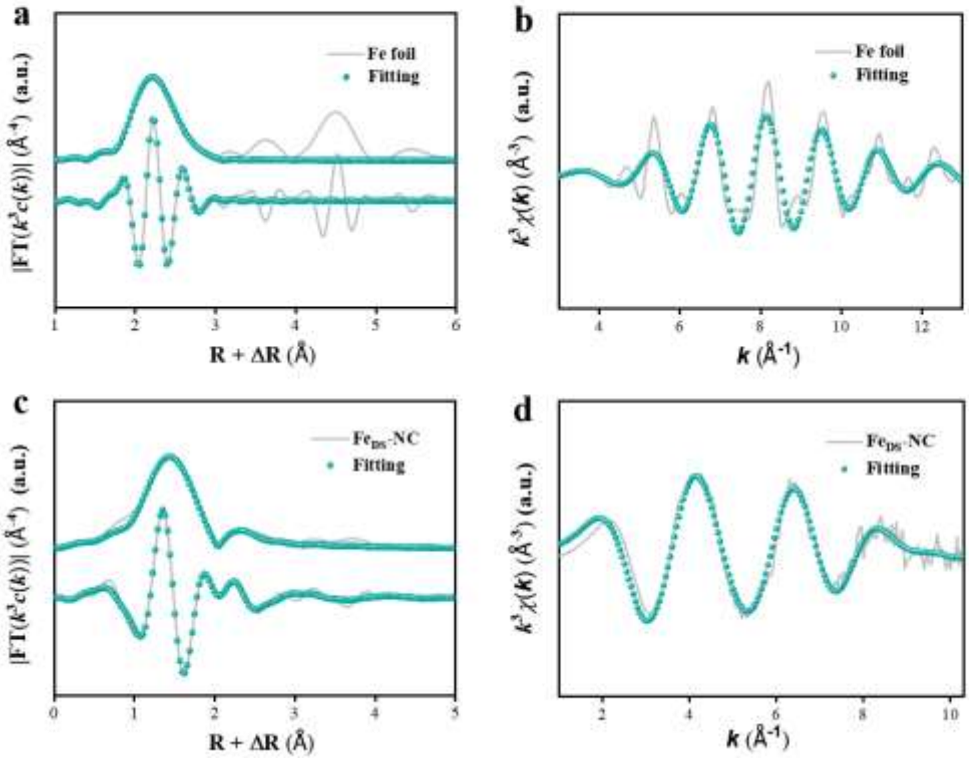

Figure S19. The FT-EXAFS fitting curves of a) Fe foil and c) Fe$_{DS}$-NC. Fe *K*-edge fitting curves in *K*-space of b) Fe foil and d) Fe$_{DS}$-NC.

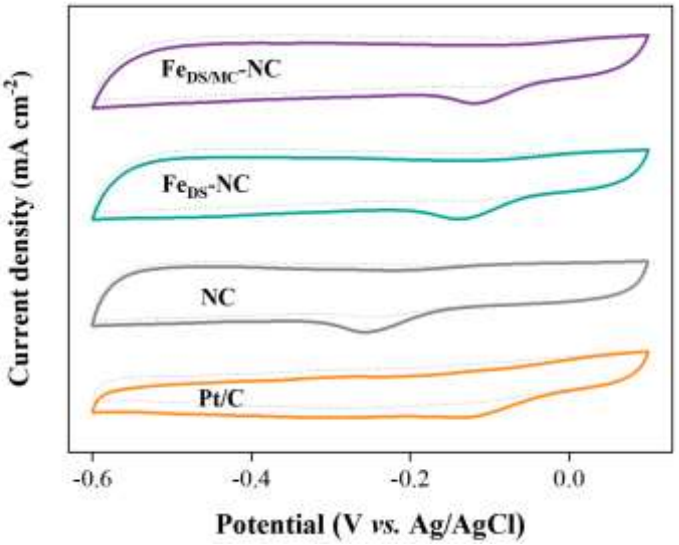

Figure S20. CV tests of samples in O_2 (solid line) or Ar (dotted line) saturated 0.1 M KOH.

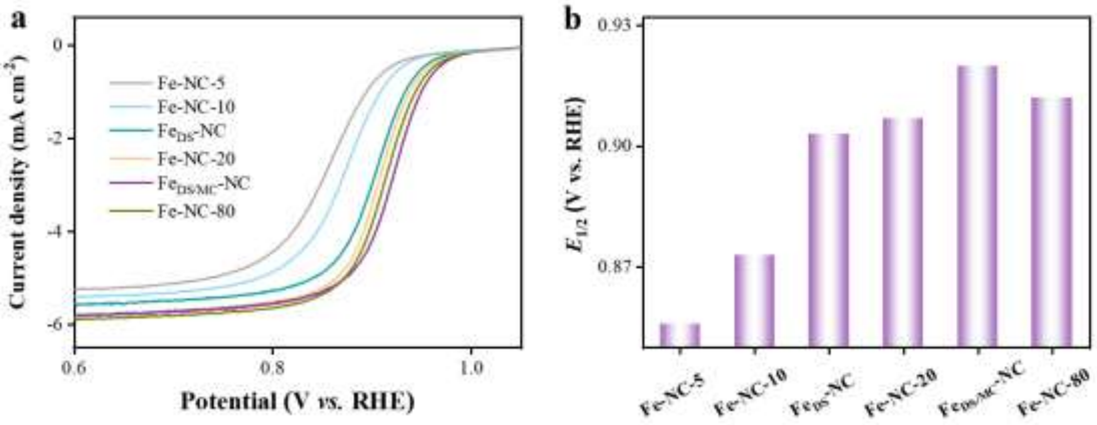

Figure S21. a) LSV curves and b) comparison of the half-wave potential for distinct samples.

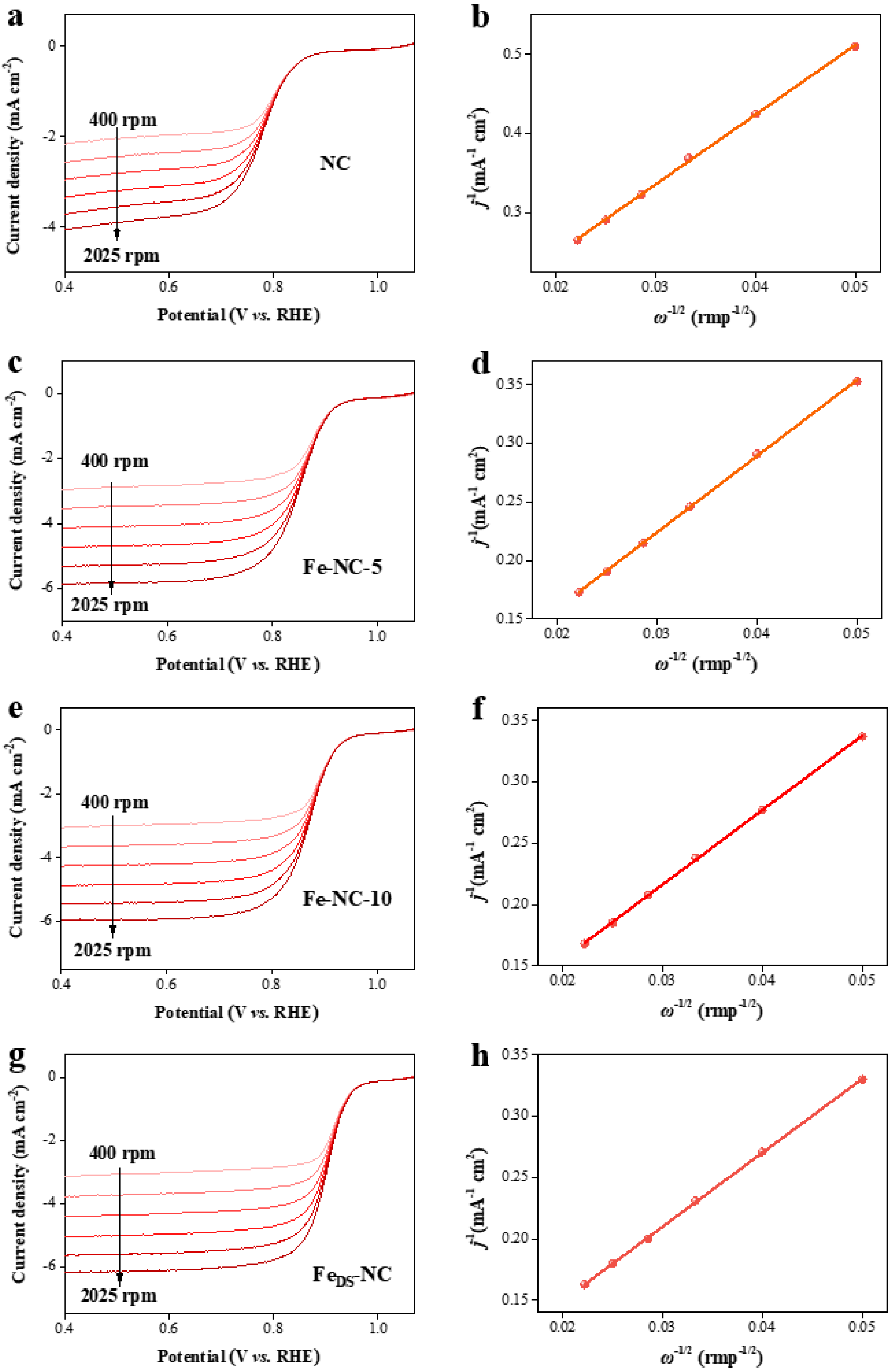

Figure S22. ORR polarization curves of samples at different rotating speeds and corresponding K-L plots.

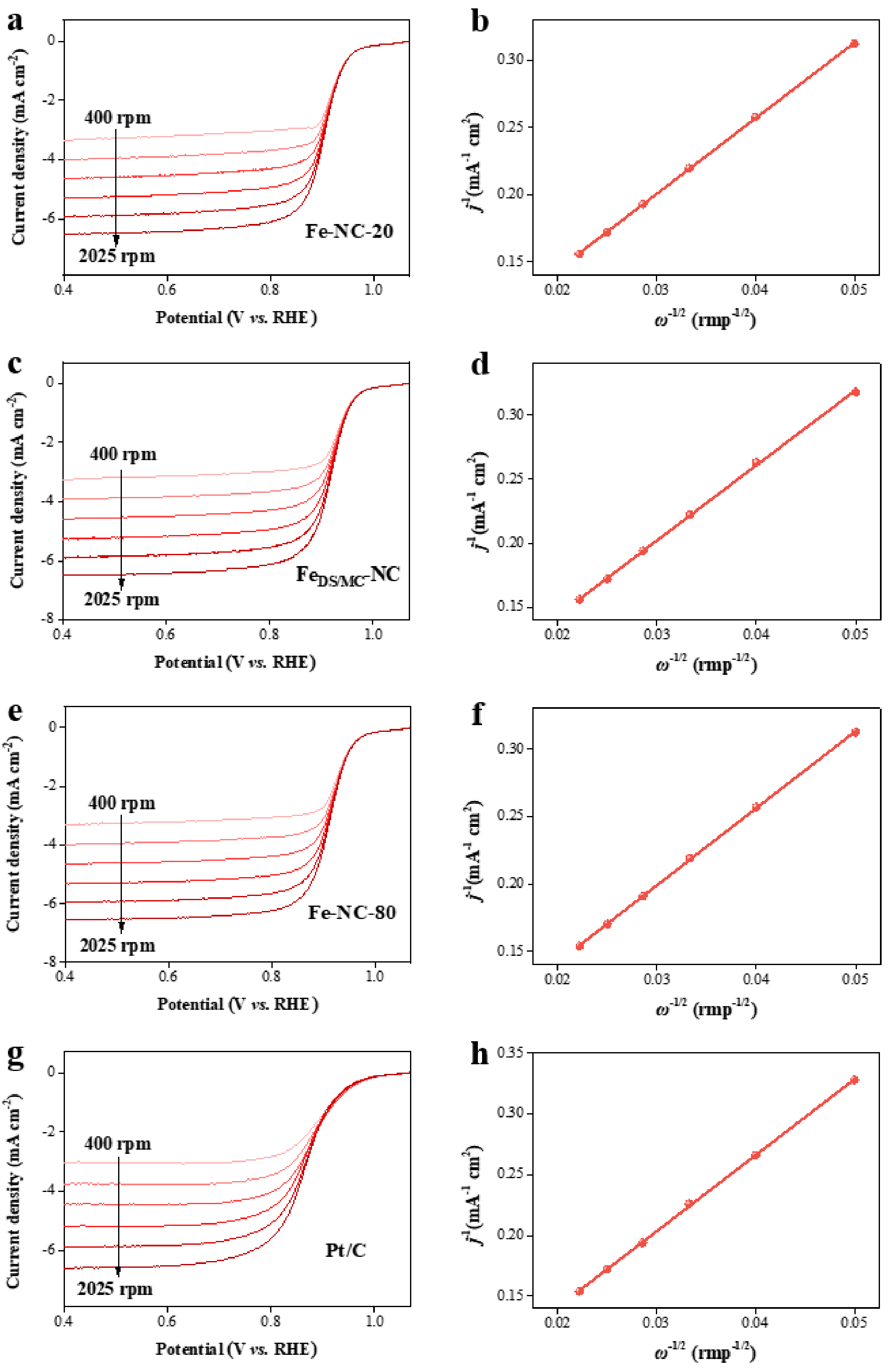

Figure S23. ORR polarization curves of samples at different rotating speeds and corresponding K-L plots.

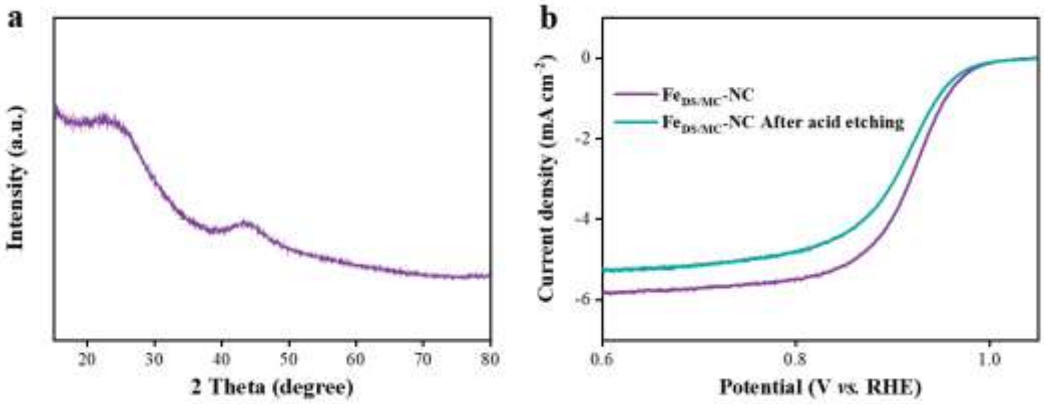

Figure S24. a) PXRD pattern and b) LSV curves of $Fe_{DS/MC}$-NC after acid etching.

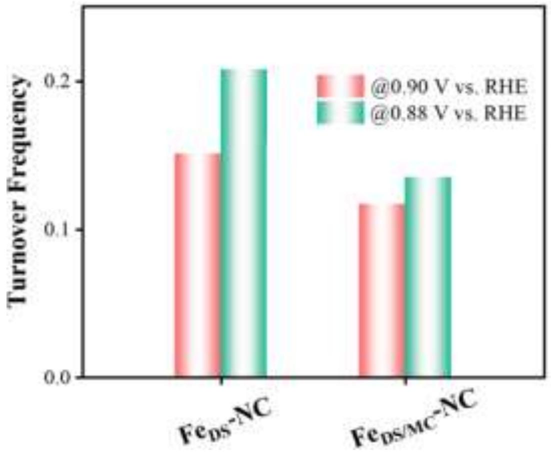

Figure S25. The TOF values of samples.

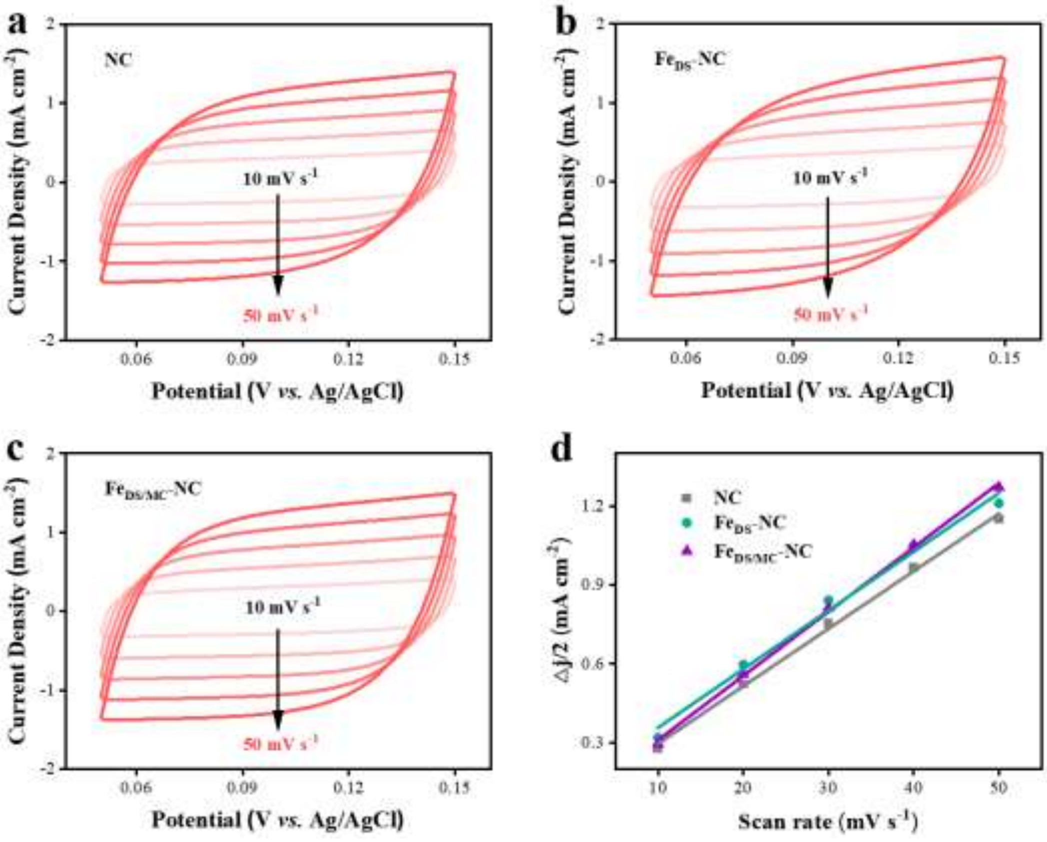

Figure S26. The CV curves of a) NC, b) Fe_{DS}-NC, and c) $Fe_{DS/MC}$-NC. d) Current density differences at various scan rates in a non-Faradaic range of samples.

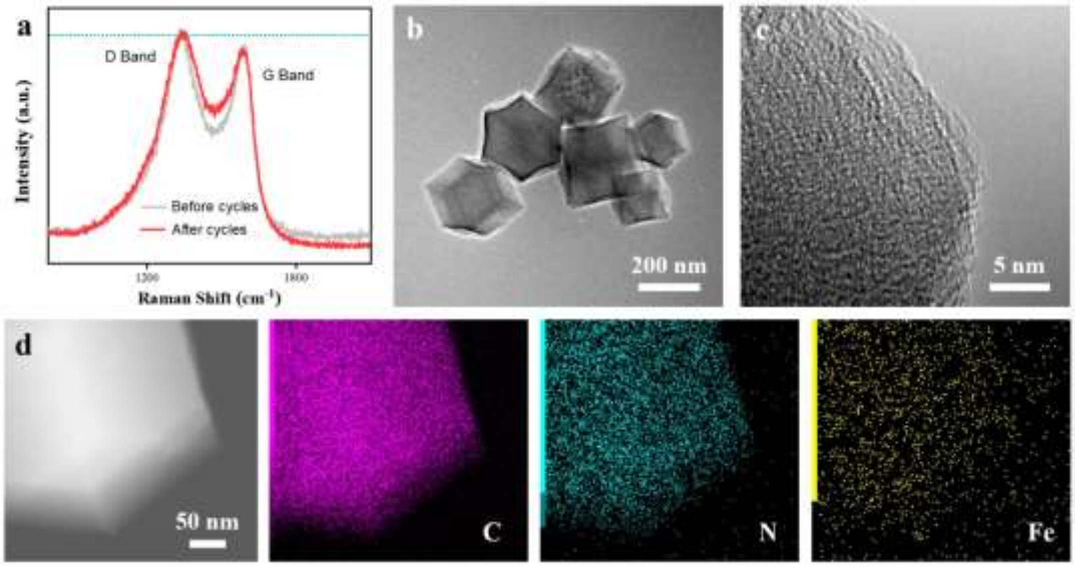

Figure S27. a) Raman spectrum of $Fe_{DS/MC}$-NC before and after cycles. b) TEM, c) HR-TEM, and d) corresponding elemental mapping images of $Fe_{DS/MC}$-NC after cycles.

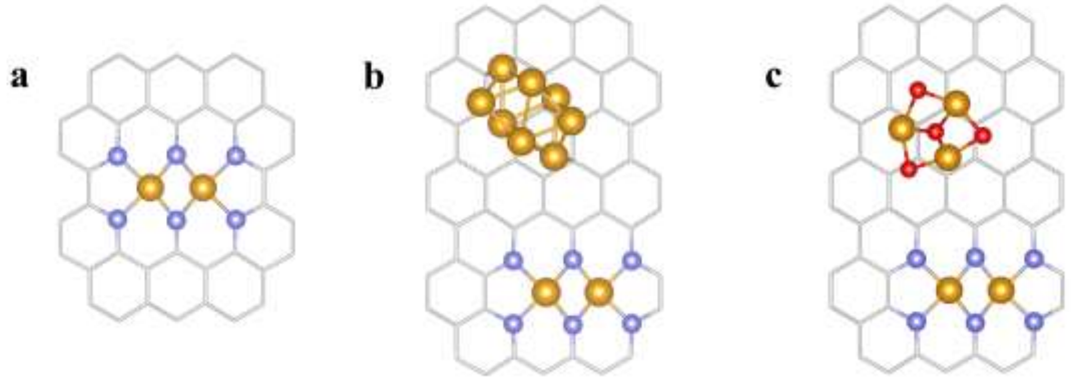

Figure S28. Structural models of a) Fe_2N_6, b) Fe_2N_6/Fe, and c) Fe_2N_6/Fe_3O_4.

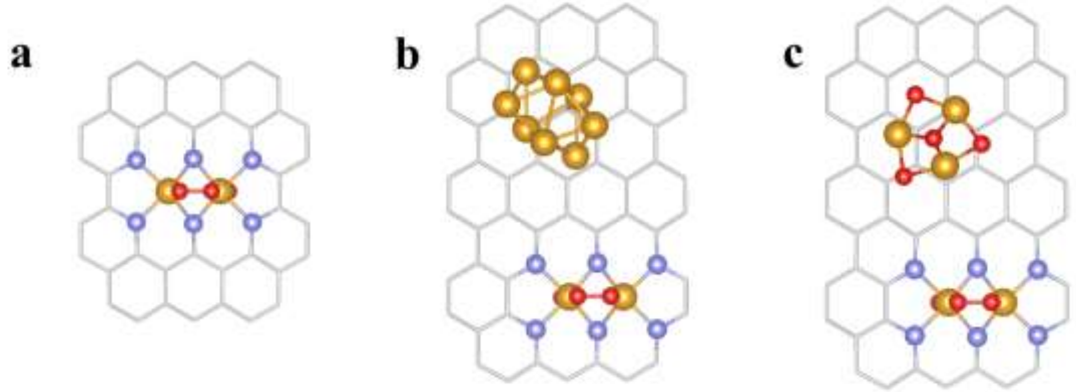

Figure S29. Structural models of a) Fe_2N_6, b) Fe_2N_6/Fe, and c) Fe_2N_6/Fe_3O_4 after oxygen adsorption.

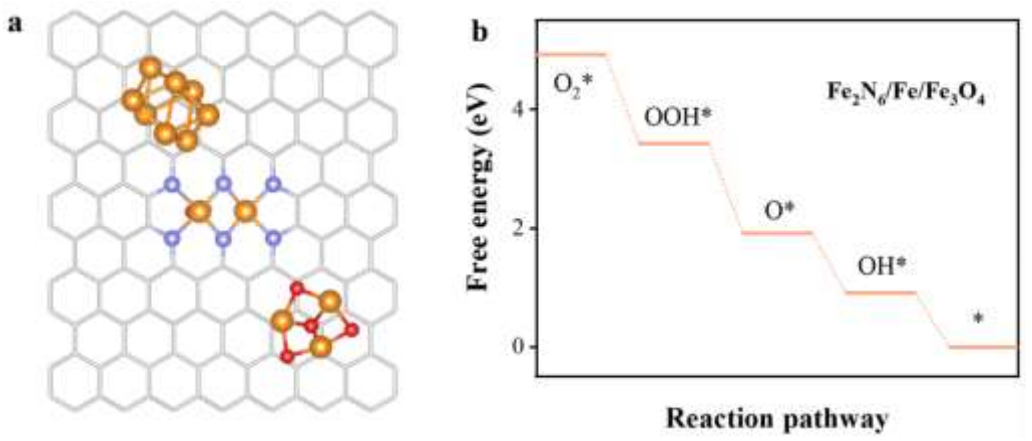

Figure S30. a) Structural models and b) the theoretical Gibbs free energy diagrams of ORR for Fe_2N_6/Fe/Fe_3O_4.

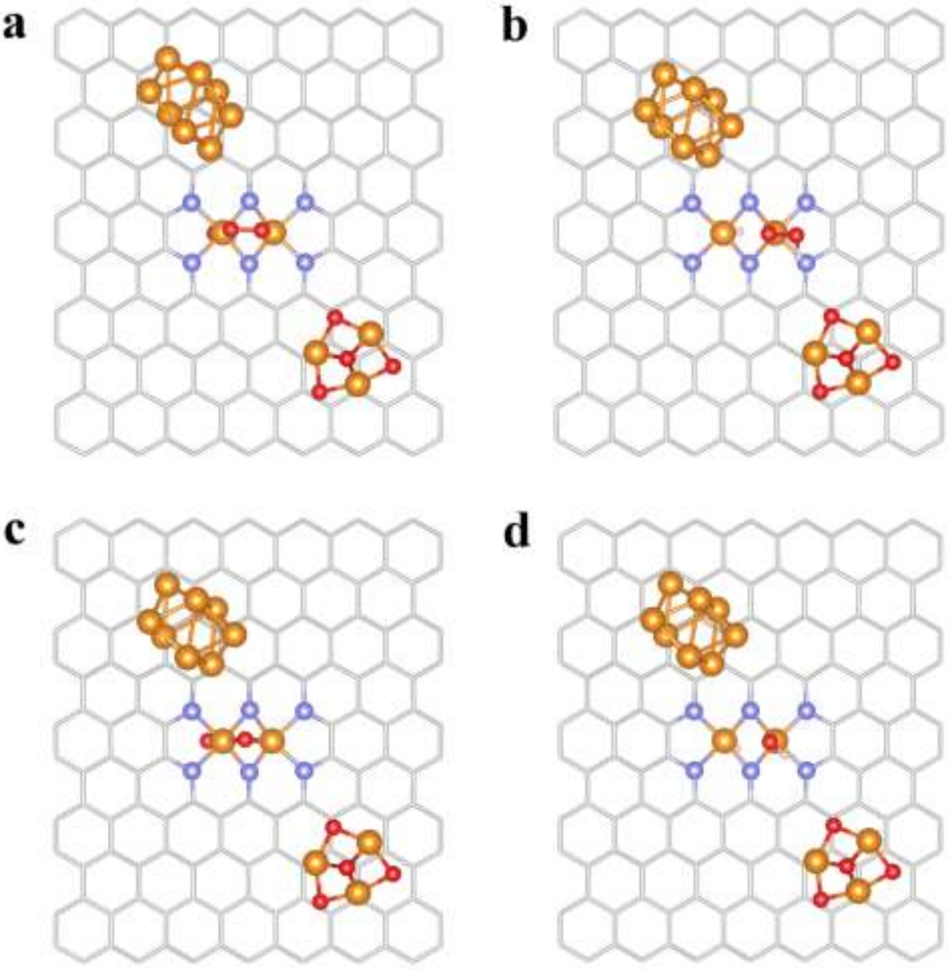

Figure S31. Optimized atomic structures for the main adsorption intermediates of $Fe_2N_6/Fe/Fe_3O_4$. a) O_2*, b) OOH*, c) O*, and d) OH*.

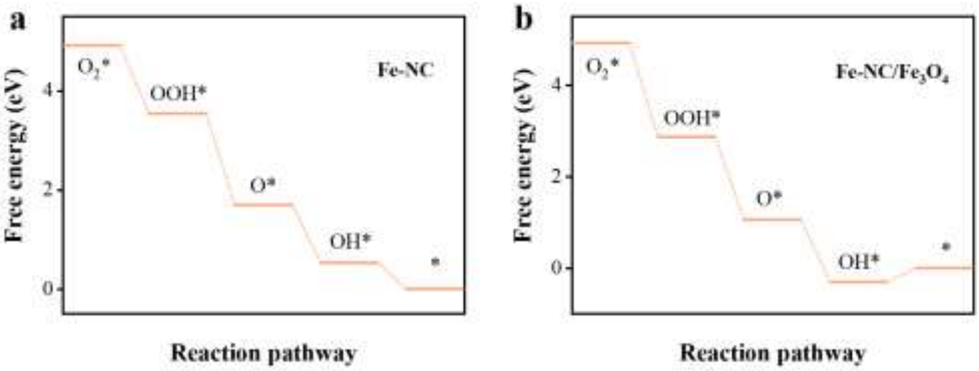

Figure S32. The theoretical Gibbs free energy diagrams of ORR for a) Fe-NC and b) Fe-NC/Fe_3O_4.

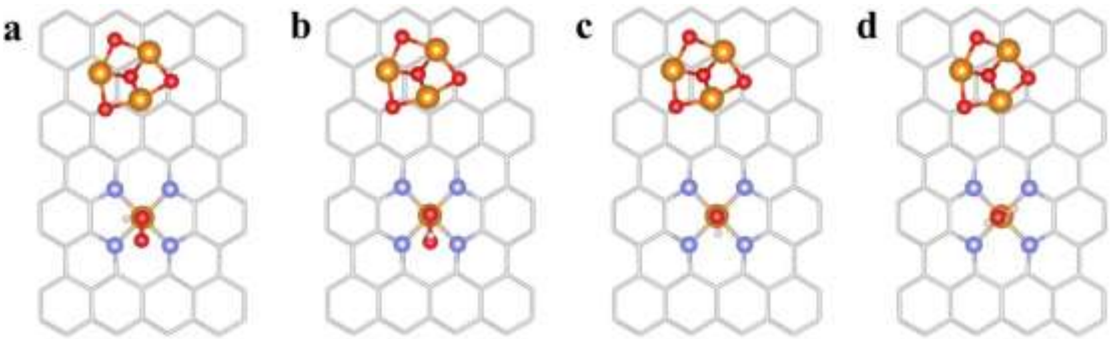

Figure S33. Optimized atomic structures for the main adsorption intermediates of Fe-NC/Fe_3O_4. a) O_2*, b) OOH*, c) O*, and d) OH*.

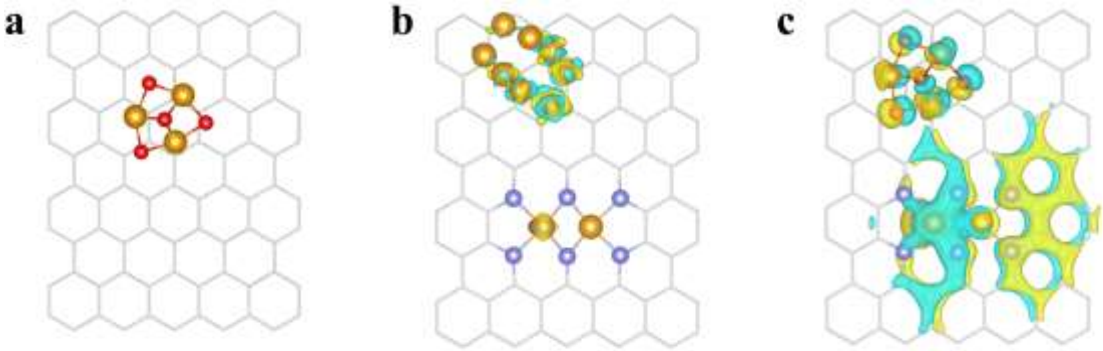

Figure S34. The differential charge density distributions of a) graphene/Fe_3O_4, b) Fe_2N_6/Fe, and c) Fe_2N_6/Fe_3O_4.

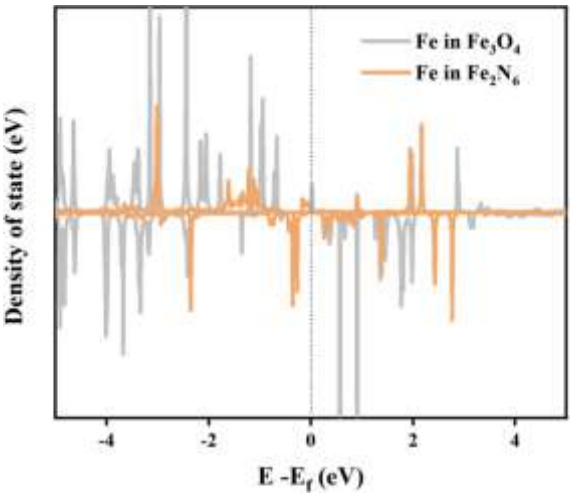

Figure S35. Projected density of states of the Fe 3*d* in Fe_3O_4 and Fe_2N_6 for Fe_2N_6/Fe_3O_4 model without OH* pre-adsorption.

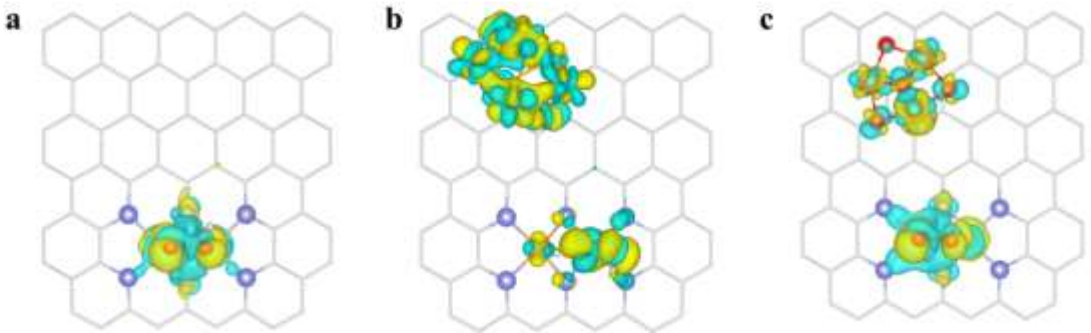

Figure S36. The differential charge density distributions of a) Fe_2N_6, b) Fe_2N_6/Fe, and c) Fe_2N_6/Fe_3O_4 after *OOH adsorption.

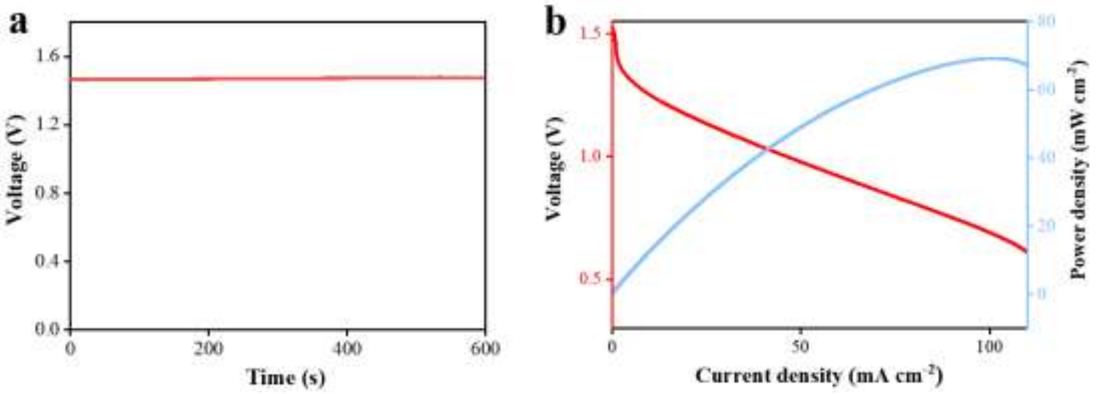

Figure S37. a) The open-circuit voltage, b) discharge polarization, and power density curves of all-solid-state rechargeable Zn-air battery with $Fe_{DS/MC}$-NC as air cathode.

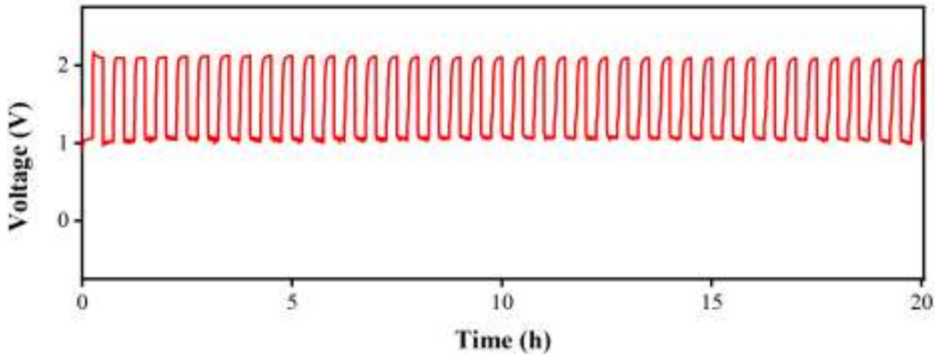

Figure S38. The cycling charge/discharge capability for the all-solid-state battery assembled with $Fe_{DS/MC}$-NC at 2 mA cm^{-2}.

Table S1. The metal element content of the synthesized catalysts.

Sample	Fe (wt%)
Fe_{DS}-NC	1.1
$Fe_{DS/MC}$-NC	2.0

Table S2. Fe *K*-edge EXAFS fitting parameters for Fe foil and samples (S_0^2 = 0.78).

Sample	Path	[a]N	[b]R/Å	R factor
Fe foil	Fe-Fe	8*	2.47 $\pm$ 0.01	0.007
	Fe-Fe	6*	2.85 $\pm$ 0.02	
Fe_{DS}-NC	Fe-N1	2[c]	1.90 $\pm$ 0.02	0.003
	Fe-N2	2[c]	2.08 $\pm$ 0.02	
	Fe-Fe	1[c]	2.52 $\pm$ 0.20	

[a]N, coordination number; [b]R, distance between absorber and backscatter atoms; [c]These coordination numbers were constrained as N (Fe-N1 = 2), N (Fe-N2 = 2) and N (Fe-Fe = 1) based on module. R factor indicates the goodness of the fit. Error bounds (accuracies) that characterize the structural parameters obtained by EXAFS spectroscopy were estimated as N ± 20%, R ± 1%; S_0^2 was fixed to 0.78 according to the experimental EXAFS fit of Fe foil by fixing N as the known crystallographic value. Fitting range: 3.0 ≤ k (Å) ≤ 13.0 and 1.0 ≤ R (Å) ≤ 3.0 (Fe foil); 3.0 ≤ k (Å) ≤ 10.3 and 1.0 ≤ R (Å) ≤ 3.0 (Fe_{DS}-NC). A reasonable range of EXAFS fitting parameters: 0.700 < S_0^2 < 1.000; N > 0; R factor < 0.02.

Table S3. Comparison of ORR performance in 0.1 M KOH for similar catalysts.

Catalyst	Tafel slopes (mV dec^{-1})	$E_{1/2}$ [a)] (V)	E_O [b)] (V)	Mass loading (mg cm^{-2})	Reference
Fe_2DAC	57	0.898	N/A[c)]	0.57	*Angew. Chem., Int. Ed.* **2023**, *62*, 202304412.
Ni,Fe-DSAs/NCs	64	0.895	N/A	0.3	*ACS Nano* **2023**, *17*, 8622.
$FeMn_{ac}$/Mn-N_4C	87.4	0.9	1	0.8	*Angew. Chem., Int. Ed.* **2023**, 62, 202214988.
Fe_2S_2@CN	74	0.92	1.07	N/A	*Angew. Chem., Int. Ed.* **2023**, *62*, 202300826.
Fe_5-Cu-N-mC	55.3	0.92	N/A	0.3	*Angew. Chem., Int. Ed.* **2023**, *62*, 202308344.

NiFe-LDH/Fe_1-N-C	60	0.9	1	0.3	*Adv. Energy Mater.* **2023,** *13*, 2203609.
Fe/NCFs-NH_3	70.82	0.89	1.02	0.383	*Adv. Mater*. **2022**, *34*, 2105410.
Fe_2@PDA-ZIF-900	47	0.951	N/A	0.5102	*Adv. Funct. Mater.* **2022**, *32*, 2205637.
Fe_1Co_3-NC-1100	69.06	0.877	1.05	0.256	*ACS Catal.* **2022**, *12*, 1216.
Fe-ACSA@NC	78	0.9	1.03	0.26	*Angew. Chem., Int. Ed.* **2022**, *61*, 202116068
Fe SAs-Fe_2P NPs/NPCFs-2.5	45.4	0.91	1.03	N/A	*Adv. Mater.* **2022**, *34*, 2203621.
FeMn-DSAC	33	0.922	1.04	0.3	*Angew. Chem., Int. Ed.* **2022**, *61*, 202115219.
FeCo-DACs/NC	85	0.87	0.98	0.26	*Adv. Mater*. **2022**, *34*, 2107421
Fe/Zn-N-C	47	0.906	N/A	0.38	*Energy Environ. Sci.* **2022**, *15*, 1601.
Fe SA-NSC	59	0.86	0.94	0.1	*ACS Energy Lett*. **2021**, *6*, 379.
ZnCoFe-N-C	N/A	0.878	0.95	0.71	*ACS Appl. Mater. Interfaces* **2021**, 24, 28324.
Fe_1-$NS_{1.3}$C	N/A	0.86	0.97	N/A	*Angew. Chem., Int. Ed*. **2021**, *60*, 25404.
FeCo-N-HCN	52.1	0.86	0.98	0.10	*Adv. Funct. Mater.* **2021**, *31*, 2011289.
MoS_2@Fe-N-C NSs	84.7	0.84	N/A	N/A	*Proc. Natl. Acad. Sci. U.S.A.* **2021,** *118*, e2110036118.
Ni-N_4/GHSs/Fe-N_4	55	0.83	0.93	0.26	*Adv. Mater.* **2020**, *32*, 2003134.
Fe/N-G-SAC	50	0.89	N/A	0.6	*Adv. Mater.* **2020**, *32*, 2004900.
Fe-SAs/NPS-HC	36	0.912	N/A	N/A	*Nat Commun.* **2018**, *9*, 5422.
Fe_{AC}@Fe_{SA}-N-C	61	0.912	N/A	0.37	*ACS Nano* **2019**, *13*, 11853.
Fe_3O_4@FeNC	58.8	0.890	1.007	N/A	*Carbon* **2020**, *162*, 245.
Fe/Meso-NC-1000	56	0.88	1.00	0.34	*Adv. Mater*. **2022**, *34*, 2107291.
Fe@C-FeNC	64	0.917	1.025	0.239	*Nano-Micro Lett.* **2023**, *15*, 48.
Fe@FeHPNC-P2	65.31	0.88	N/A	0.459	*J. Energy Storage* **2024**, *96*, 112672.
Fe_{SA}/Fe_{AC}-NC	56.89	0.88	0.96	N/A	*J. Energy. Chem.* **2025**, *102*, 413.

$Fe_{SA+clusters}$	50	0.85	0.96	0.685	*Small* **2025**, *21*, 2409474.
FeSA-Fe_3C/NC	82	0.902	1.007	0.4	*Angew. Chem., Int. Ed.* **2025**, *64*, e202501266.
Fe_2N_{nc}/Fe_1-N-C	54.2	0.957	1.105	0.42	*Angew. Chem., Int. Ed.* **2025**, *64*, e202504935.
Fe_{SA}/Fe_{AC}@PPy/C C	75.0	0.83	0.97	N/A	*Energy Environ. Sci.* **2025**, *18*, 2839.
**$Fe_{DS/MC}$-NC-40**	**56.1**	**0.920**	**0.990**	**0.26**	**This Work**

a) half-wave potential; b) onset potential; c) not available

Table S4. The calculated oxygen adsorption energies for different catalytic models.

**Models**	**Adsorption energy (eV)**
Fe_2N_6	-0.89
Fe_2N_6/Fe	0.08
Fe_2N_6/Fe_3O_4	-0.91

Table S5. The calculated *d*-band center values for different theoretical models.

**Models**	***d* band center (eV)**	
	**Spin up**	**Spin down**
Fe_2N_6	-1.554	-1.773
Fe_2N_6/Fe	-1.697	-1.416
Fe_2N_6/Fe_3O_4	-1.978	-0.955

Table S6. Comparison of Zn-air battery performance for similar catalysts.

**Catalyst**	**OCV**	**Maximum power density ($mW\ cm^{-2}$)**	**Rechargeability ($mA\ cm^{-2}$)**	**References**
Fe_2N_6-S	1.49	200.1	30 min/cycle for 400 h (10 $mA\ cm^{-2}$)	*Adv. Mater.* **2024**, *36*, 2309231.
$FeMn_{ac}/Mn-N_4C$	1.46	207	10 min/cycle for 100 h (5 $mA\ cm^{-2}$)	*Angew. Chem., Int. Ed.* **2023**, *62*, 202214988.

Fe_5-Cu-N-mC	1.48	214.8	-- for 240 h (5 mA cm^{-2})	*Angew. Chem., Int. Ed.* **2023**, *62*, 20230834.
NiFe-LDH/Fe_1-N-C	1.54	205	-- for 400 h (2 mA cm^{-2})	*Adv. Energy Mater.* **2023,** *13*, 2203609.
Ni,Fe-DSAs/NCs	1.491	217.5	240 min/cycle for 500 h (5 mA cm^{-2})	*ACS Nano* **2023**, *17*, 8622.
FeCo-DACs/NC	1.50	175	30 min/cycle for 480 cycles (10 mA cm^{-2})	*Adv. Mater*. **2022**, *34*, 2107421.
3D SAFe	1.47	156	80 h (10 mA cm^{-2})	*Nano Lett*. **2022**, *22*, 7386.
CoFe-N-C	1.49	142.1	30 min/cycle for 200 h (5 mA cm^{-2})	*Nano Lett.* **2022**, *22*, 3392.
FeN_4-O-NC	1.51	214.2	N/A[a)]	*Adv. Mater.* **2022**, *34*, 202544.
Fe_1Co_1-CNF	1.45	201.7	20 min/cycle for 210 h (20 mA cm^{-2})	*Nano Energy*. **2021**, *87*, 106147.
Fe,Mn/N-C	N/A	160.8	-- for 81 h (5 mA cm^{-2})	*Nat. Commun.* **2021**, *12*, 1734.
Fe/Ni-N_x/OC	1.525	148	300 cycles (20 mA cm^{-2})	*Adv. Mater*. **2020**, *32*, 2004670.
Fe@Fe_{SA}-N-C-900	N/A	110	--for 500 h	*J. Energy Chem.* **2021**, *61*, 612.
FeCo/FeCoNi@NCNTs-HF	1.481	156.22	40 min/cycle for 120 h (10 mA cm^{-2})	*Appl. Catal. B-Environ.* **2019**, *254*, 26.
Ni-N_4/GHSs/Fe-N_4	1.45	N/A	600 cycles (10 mA cm^{-2})	*Adv. Mater.* **2020**, *32*, 2003134.
Co_{40}SAs/AC@NG	1.5	221	10 min/cycle for 400 cycles (10 mA cm^{-2})	*Adv. Funct. Mater.* **2023**, *33*, 2209726.
Co-SAs/SNPs@NC	1.493	223.5	40 min/cycle for 720 h (5 mA cm^{-2})	*Adv. Funct. Mater.* **2021**, *31*, 2104735.
**$Fe_{DS/MC}$-NC**	**1.52**	**214.6**	**20 min/cycle for 1000 cycles (10 mA cm^{-2})**	**This work**

References

[1] M. Liu, X. Wang, S. Cao, X. Lu, W. Li, N. Li, X. H. Bu, *Adv. Mater.* **2024**, *36*, 2309231.

[2] G. Kresse, J. Furthmüller, *Phys. Rev. B* **1996**, *54*, 11169.

[3] J. P. Perdew, K. Burke, M. Ernzerhof, *Phys. Rev. Lett.* **1996**, *77*, 3865.

[4] K. Mathew, R. Sundararaman, K. Letchworth-Weaver, T. A. Arias, R. G. Hennig, *J. Chem. Phys.* **2014**, *140*, 084106.

[5] J. K. NøRskov, J. Rossmeisl, A. Logadottir, L. Lindqvist, J. R. Kitchin, T. Bligaard, H. Jónsson, *J. Phys. Chem. B* **2004**, *108*, 17886.

[6] Y. Zheng, Y. Jiao, Y. Zhu, Q. Cai, A. Vasileff, L. H. Li, Y. Han, Y. Chen, S. Z. Qiao, *J. Am. Chem. Soc.* **2017**, *139*, 3336.
